# Tough hydrogel-coated containment capsule of magnetic liquid metal for remote gastrointestinal operation

**DOI:** 10.1093/nsr/nwaf042

**Published:** 2025-02-11

**Authors:** Yifeng Shen, Jiasheng Cao, Enjie Zhou, Lei Wang, Kaihang Zhang, Yaoting Xue, Hui Yuan, Jiahao Hu, Siyang Li, Zhikun Miao, Yukai Zhao, Tuck-Whye Wong, Tiefeng Li, Mingyu Chen, Xuxu Yang, Wei Yang

**Affiliations:** Center for X-Mechanics, Department of Engineering Mechanics, Zhejiang University, Hangzhou 310027, China; Department of Engineering Mechanics, Zhejiang University, Hangzhou 310027, China; Department of General Surgery, Sir Run-Run Shaw Hospital, Zhejiang University, Hangzhou 310016, China; Zhejiang University School of Medicine, Zhejiang University, Hangzhou 310058, China; Department of General Surgery, Sir Run-Run Shaw Hospital, Zhejiang University, Hangzhou 310016, China; Zhejiang University School of Medicine, Zhejiang University, Hangzhou 310058, China; Center for X-Mechanics, Department of Engineering Mechanics, Zhejiang University, Hangzhou 310027, China; Department of Engineering Mechanics, Zhejiang University, Hangzhou 310027, China; Center for X-Mechanics, Department of Engineering Mechanics, Zhejiang University, Hangzhou 310027, China; Department of Engineering Mechanics, Zhejiang University, Hangzhou 310027, China; Center for X-Mechanics, Department of Engineering Mechanics, Zhejiang University, Hangzhou 310027, China; Department of Engineering Mechanics, Zhejiang University, Hangzhou 310027, China; Center for X-Mechanics, Department of Engineering Mechanics, Zhejiang University, Hangzhou 310027, China; Department of Engineering Mechanics, Zhejiang University, Hangzhou 310027, China; Department of General Surgery, Sir Run-Run Shaw Hospital, Zhejiang University, Hangzhou 310016, China; Zhejiang University School of Medicine, Zhejiang University, Hangzhou 310058, China; Center for X-Mechanics, Department of Engineering Mechanics, Zhejiang University, Hangzhou 310027, China; Department of Engineering Mechanics, Zhejiang University, Hangzhou 310027, China; Center for X-Mechanics, Department of Engineering Mechanics, Zhejiang University, Hangzhou 310027, China; Department of Engineering Mechanics, Zhejiang University, Hangzhou 310027, China; Center for X-Mechanics, Department of Engineering Mechanics, Zhejiang University, Hangzhou 310027, China; Department of Engineering Mechanics, Zhejiang University, Hangzhou 310027, China; School of Biomedical Engineering and Health Sciences and Advanced Membrane Technology Research Centre, Universiti Teknologi Malaysia, Skudai 81310, Malaysia; Center for X-Mechanics, Department of Engineering Mechanics, Zhejiang University, Hangzhou 310027, China; Department of Engineering Mechanics, Zhejiang University, Hangzhou 310027, China; Department of General Surgery, Sir Run-Run Shaw Hospital, Zhejiang University, Hangzhou 310016, China; Zhejiang University School of Medicine, Zhejiang University, Hangzhou 310058, China; Center for X-Mechanics, Department of Engineering Mechanics, Zhejiang University, Hangzhou 310027, China; Department of Engineering Mechanics, Zhejiang University, Hangzhou 310027, China; Center for X-Mechanics, Department of Engineering Mechanics, Zhejiang University, Hangzhou 310027, China; Department of Engineering Mechanics, Zhejiang University, Hangzhou 310027, China

**Keywords:** containment capsule, magnetic liquid metal, hydrogel coating, targeted thermal ablation

## Abstract

Gallium-based liquid metals, when combined with magnetic agents, emerge as intelligent materials with potential applications in soft robotics within biomedical engineering. However, concerns have arisen from the residual presence of liquid metal, raising long-term biological risks. Herein, we propose a containment method that involves the rolling of magnetic liquid-metal droplets in lyophilized powders, resulting in the formation of intact hydrogel coatings upon hydration. These hydrogel coatings adhere to the liquid-metal surface, forming a cohesive network through hydrogen bonding between carboxylic acid groups and siloxane linkages from silanol groups. This synergy of physical and chemical interactions enables hydrogel coatings with exceptional stretchability, fracture energy and interfacial bonding to liquid metals. Consequently, the hydrogel-coated containment capsule of magnetic liquid metal exhibits remarkable resilience to cyclic compression, enduring strains of ≤85%, while also withstanding impacts from heights of >14 m. Moreover, the containment capsules demonstrate large deformation capabilities, dexterous locomotion and wireless heating under the control of static and alternating magnetic fields. They showcase the capability for remote thermal ablation operations on *ex vivo* porcine stomachs and *in vivo* rabbit models.

## Teaser

Self-healing lyophilized hydrogel powders create a tough coating on magnetic liquid metals through rolling and hydrating, enabling effective containment and offering great potential for capsule robots in remote thermal ablation operation.

## INTRODUCTION

**Figure 1.**
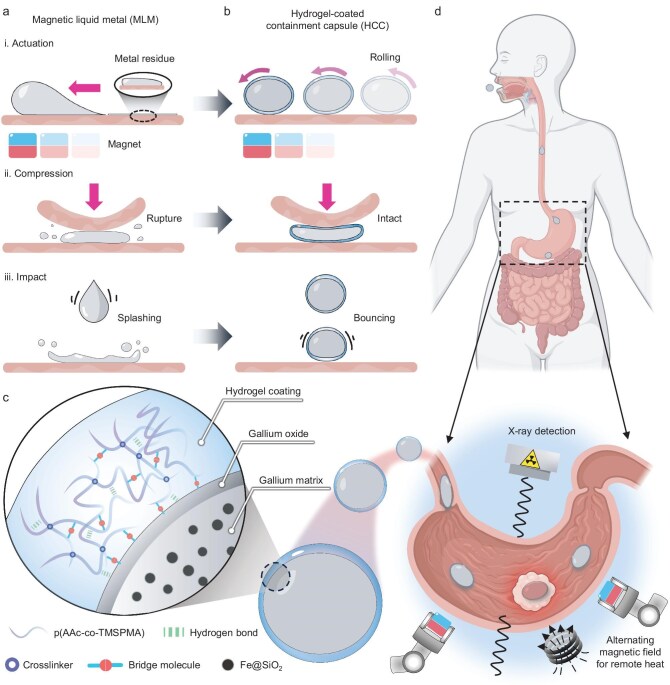
Concept of HCC of MLM and schematic of remote theragnostic gastrointestinal operation using HCC. (a) Mechanical instability of MLM droplet without containment. (b) Mechanical reliability of HCCs during actuation, compression and impact. (c) Details about the compositional structure of HCCs. (d) Schematic illustration of remotely targeted thermal ablation operation in stomach models using HCCs manipulated by magnetic robotic arm under the guidance of X-ray imaging. Image (d) created with BioRender.com, with permission.

Gallium-based liquid metals are highly promising for biomedical engineering due to their compliance, conductivity, biocompatibility, imaging feasibility and antibacterial properties [1–4] compared with other flexible matters (details in Table S1). When further integrated with magnetic agents, these functionalized liquid metals can serve as miniature soft robots that are controlled by external magnetic fields [5–8]. These robots are particularly soft, allowing them to access biological tissue non-invasively and to conduct medical operations [9,10], such as targeted magnetic ablations [11]. Compared with common thermoseeds (details in Table S2), magnetic liquid metals (MLMs) possess higher heating efficiency due to the combined effects of their liquid-state conformability, magnetothermal properties from magnetic agents and eddy-current heating arising from their metallic nature [12–14]. However, native oxide films on the surface of liquid metals are susceptible to adherence to tissues [15]. Moreover, the collection of scattered liquid-metal droplets is challenging due to their high fluidity and surface tension [16] (Fig. 1a). Accordingly, a serious concern exists over the issue of leaving metal residues inside the body, potentially causing long-term health risks [17–19].

Encapsulation creates a protective barrier around liquid metals, preventing leakage while maintaining their functionalities. On a microscopic scale, liquid metals have been encapsulated within ligand molecules [20,21], polymers [22,23] and inorganic matters [24–26] to form micro/nanoparticles. However, maneuvering and monitoring these encapsulated particles during medical procedures are challenging [27], which could limit therapeutic effectiveness and pose risks of residual material. Alternatively, liquid-metal particles are capable of dispersing in elastomers [28–31], but the composites with high concentrations of liquid-metal particles can leak metal when deformed [32]. Practical approaches involve encapsulating larger amounts of liquid metal on a macroscale by using thin films, bulky silicone molds or 3D-printed rigid shells (details in Table S3) [33–35]. Nevertheless, these containment methods compromise the flexibility of liquid metal and cause a mismatch in mechanical properties with biological tissues, potentially leading to mechanical damage [36]. The use of a dip-coating method for surface encapsulation requires the precursor solution to wet the surface and uneven coating thickness on the bulk material often occurs due to the influence of gravity. The development of liquid-metal marbles by coating droplets with solid particles such as graphene [37], metal oxides [38], poly(tetrafluoroethylene) [39], fluorescent nanoparticles [40] and metal layers [41] presents an alternative strategy. The coating, formed through rolling and adhesion, prevents direct contact between the liquid metal and the substrates, imparts elasticity while maintaining the inherent softness of the liquid metals. Yet, the existing coating layers are incompact and fragile, leaving them unsuitable for long-term use or exposure to strong impacts, which can result in liquid-metal leakage as the particles detach [42]. As a remedy, a soft, thin but tough containment coating is desired.

Here, a rolling approach is proposed to coat a thin and tough hydrogel on the MLM, thus effectively preventing leakage while ensuring its softness and utility in biomedical applications. This approach enables the rapid encapsulation of spherical liquid metal, achieving a hydrogel coating with relatively uniform thickness, continuous coverage and robust interfacial adhesion. Similar to the craft of making rice glue balls, moist frozen MLM droplets are rolled in lyophilized hydrogel powders (LHPs) to construct a tough hydrogel coating for containment. This encapsulation allows MLM droplets to resist violent compression and impacts without rupture or leaving metal residues behind (Fig. 1b). The exceptional mechanical properties of the hydrogel coating are attributed to the synergy of physical and chemical interactions. Specifically, the polymer network within the hydrogel generates numerous hydrogen bonds and fraction siloxane linkages upon hydrating. They link separate discrete LHPs into a tough hydrogel coating and bridge the hydrogel to the liquid-metal surface (Fig. 1c). The flexibility of the hydrogel coating ensures that the containment system retains the inherent softness of liquid-metal droplets. Furthermore, the fabricated hydrogel-coated containment capsule (HCC) of MLM can perform dexterous locomotion and wireless heating, while also being feasible to be monitored by X-ray during medical operations. These capabilities facilitate remotely targeted thermal ablation operations (Fig. 1d), as validated through experiments with *ex vivo* porcine stomachs and *in vivo* rabbit models. The intact integrity of containment guarantees that no liquid metal leaks into the body during ingestion and manipulation. This effective containment strategy for multifunctional liquid metals opens up new possibilities for their safe use in clinical applications.

## RESULTS

### Fabrication and characterization of HCC

We encapsulated MLMs within a tough hydrogel coating by using a simple method inspired by the traditional craft of making rice glue balls (Fig. S1a). This preparation process involved rolling moist and frozen MLM balls in LHPs, followed by hydrating LHPs by spraying with an aqueous solution (Fig. S1b). As for the inner ingredients, MLM droplets were prepared by mixing magnetic agents with gallium matrices (Fig. S2). These magnetic agents were iron microparticles modified with silica shells (Fe@SiO_2_) (Fig. S3). The silica shells effectively prevent alloying and chemical corrosion (Figs S3d and S4) [43]. Moreover, the LHPs were fabricated by freeze-drying and grinding bulky hydrogels, which were synthesized from a copolymer of acrylic acid (AAc) and 3-(trimethoxysilyl)propyl methacrylate (TMSPMA). These powders, with an average size of 90.9 μm, featured porous structures that facilitated rapid hydration (Fig. S5).

**Figure 2.**
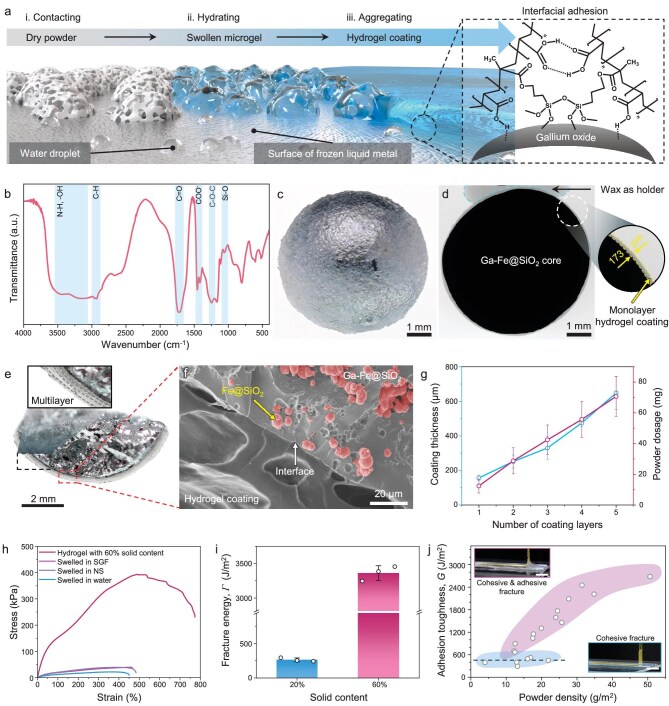
Self-gelling mechanism and characteristics of hydrogel coating on HCCs. (a) Schematic illustration of the self-gelling process from lyophilized hydrogel powders (LHPs) to an intact hydrogel coating. The insert on the right shows the role of Si–O–Si bonds and hydrogen bonds in promoting interfacial adhesion. (b) Fourier-transform infrared spectroscopy for LHPs, where absorption peaks in blue shaded areas represent the existence of chemical groups and bonds. (c) Optical image presenting the appearance of the fully hydrated 1-HCC. (d) X-ray image showing the cross section of 1-HCC. The black areas correspond to the high-density Ga-Fe@SiO_2_. The gray areas around the black sphere represent the low-density hydrogel coating and the gray areas in the blue dashed box represent the low-density wax as a holder. The zoomed-in image highlights the local thickness of the hydrogel coating. (e) Optical image showing a 4-HCC with dried hydrogel coatings. The zoom-in image shows multilayer hydrogel structures. (f) Scanning electron microscope image showing the compact interface between the hydrogel coating and the MLM, and the distribution of inner Fe@SiO_2_ microparticles. (g) Linear relationship between coating thickness and powder dosage with the number of coating layers. Data are presented as mean ± SD; *n* = 3 independent experiments. (h) Stress–strain curves of hydrogels hydrated to 60% solid content and those swelled in the SGF, NS and pure water. (i) Fracture energies of hydrogels hydrated to 20% solid content and 60% solid content. Data are presented as mean ± SD; *n* = 3 independent experiments. (j) Interfacial toughness between hydrogel coating and MLM layer exceeds 400 J/m^2^. Scale bars, 2 cm.

During the containment fabrication process, the formation of the continuous hydrogel coating involves three stages (Fig. 2a), which develop through the self-gelling of discrete LHPs in the presence of moisture. Firstly, the LHPs come into contact with and adhere to the moist MLM surface during the rolling process (Stage i). This contact leads to the absorption of superficial water by the LHPs, making them sticky and facilitating preliminary adhesion. Secondly, the adhered LHPs undergo further hydration by spraying glycerol aqueous solutions (Stage ii), causing their gradual transformation into microgels. These microgels exhibit a significant number of exposed functional groups along the polymer chains of p(AAc-*co*-TMSPMA). Finally, the microgels swell further, enabling contact between them, and subsequently aggregate to form hydrogel coatings (Stage iii). The presence of numerous carboxylic acid groups (–COOH) and silanol groups (–Si–OH) within the polymer chains enabled the formation of hydrogen bonds and siloxane linkages [44,45] (Fig. 2b), which promote the mechanical integrity and stability of the hydrogel coatings. We note that the hydrogen bonds form instantly but can cleave upon attack by excess water molecules, while siloxane linkages develop gradually but remain stable for a long time [46]. Furthermore, the timeline for the formation of the hydrogel coating shows that the swift absorption of water leads to the formation of a stable hydrogel coating within minutes (Fig. S6a and Movie S1). The transformation of rheological performance also verifies the self-gelation process of the LHPs [47] (Fig. S6b), where the storage modulus (G’) curve intersects with the loss modulus (G’’) curve within ∼30 seconds after adding water. Aqueous solutions with different glycerol contents can still hydrate the LHPs to form elastic hydrogels (Fig. S7). The combination of physical and chemical interaction not only contributes to the rapid formation and stability of the hydrogel coating, but also anchors it to gallium oxides on the MLM surface for interfacial adhesion [48]. Thus, the LHPs could adhere to the surface of the MLM after rolling (appearances of balls are shown in Fig. S8) and the obtained HCCs appeared transparent after full hydration (Fig. 2c).

The hydrogel coatings exhibited continuity and robust adherence to the MLM surface. We dissected the core–shell structure of the HCC (Fig. S9) and the internal porosity of the MLM (Fig. S10) by using microcomputed tomography (micro-CT) technology. In addition to the magnetic particles that were dispersed within the MLM matrix, some air pores were also present. These pores primarily originated from air that was entrapped during the mechanical mixing process [49]. The continuous hydrogel coating seamlessly encapsulated the internally porous MLM core (Fig. 2d). Furthermore, this containment method facilitated the production of coatings with controllable thickness by regulating the number of hydrogel-coating layers. Consequently, HCCs with multilayer hydrogel coatings (termed as *n*-HCC, where *n* represents the number of hydrogel coating layers) were fabricated. A hierarchical structure of a dried hydrogel coating in 4-HCC was observed optically (Fig. 2e). The multilayer hydrogel coating exhibited a densely packed structure. The scanning electron microscope image revealed that the coatings were bonded to the MLM core with a robust interface (Fig. 2f). This robust interface adhesion was attributed to the close contact between the metal surface and the LHPs upon mechanical rolling, as well as physical and chemical interlinking during hydration. We also observed uniformly dispersed spherical Fe@SiO_2_ microparticles in the MLM matrix. Generally, the dosage of LHPs and the coating thickness were directly proportional to the number of hydrogel-coating layers, with each layer averaging ∼130 μm in thickness (Fig. 2g).

### Mechanical performance of hydrogel coating

The swelling behavior of the hydrogel coating significantly impacts its thickness and mechanical properties. Therefore, we further studied the swelling behavior of the hydrogel coatings by immersing them in normal saline (NS) and simulated gastric fluid (SGF) to assess the adaptability of these hydrogel coatings within the gastrointestinal environment (Fig. S11a and b). Initially, the hydrogel coating swelled rapidly in the first 100 minutes, after which the growth of the coating thickness slowed down, consistently with the observed relationship between moisture absorption and time (Fig. S11c). The solid content of the hydrogel stabilized at ∼20% after reaching equilibrium in NS and SGF solutions, where the solid content represents the mass fraction of the cross-linked polymer network within the overall hydrogel system. The hydrogel coatings that were immersed in NS for 1 day still reliably encapsulated the inner molten MLM during heating (Fig. S8d). We also studied the evolution of the hydrogel coatings when exposed to the ambient environment for a prolonged period. The average solid contents of the hydrogel coatings were maintained at ∼60% after exceeding 4 weeks (Fig. S12). Thus, we primarily focus on the mechanical performance of the hydrogel coating in two states: the preservation state (∼60% solid content) and the swelling state (∼20% solid content).

The hydrogel coatings that we developed are durable enough to prevent leakage of liquid metal by withstanding daily mechanical impacts, while remaining flexible and soft for safe use in the digestive tract. We characterized the mechanical properties of the hydrated hydrogel specimens (the preparation process of tensile specimens shown in Fig. S13). The hydrogel with a 60% solid content exhibited 530.3% stretchability, 397.4 kPa elastic modulus and 3362.1 J/m^2^ toughness, whereas the swelled hydrogels had ∼470% stretchability, ∼45 kPa elastic modulus and 262.9 J/m^2^ toughness (Fig. 2h and i, Supplementary Figs S14–S16 and Movie S2). The compressive stress–strain curves showed that the hydrogel samples with higher solid content possessed better robustness and compressive performance (Fig. S17). The high toughness and strength of the 60% solid content hydrogels could be attributed to the dense microscopic restraints including chain friction, hydrogen bond restraints and chain entanglements [50–52]. The moduli of the swelled hydrogels, being lower than the modulus of gastrointestinal tissue (120–150 kPa [53]), suggested that they could reduce the risk of mechanical damage. Furthermore, we measured the adhesion performance between the hydrogel coating and the MLM substrate through lap shear and tearing tests. The adhesion strength, measured at ∼25.4 kPa via the lap shear test, remained consistent regardless of the thickness of the hydrogel coating (Fig. S18). The adhesion toughness, measured via the 90°-tearing test, was ∼425.3 J/m^2^ during cohesive fracture. An adhesion toughness of 600–2600 J/m^2^ was also measured when cohesive and adhesive fracture models coexisted, which may better fit practical observations of coating failure (Fig. 2j, Fig. S19 and Movie S3).

### Mechanical reliability of HCCs

**Figure 3.**
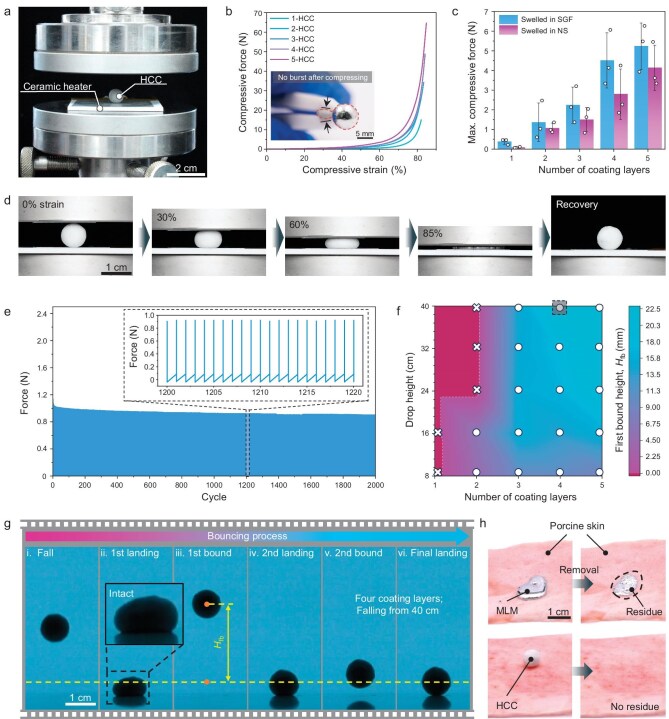
Mechanical reliability of HCCs. (a) Optical image showing a compression test conducted on an HCC while its temperature was maintained at ∼50°C by using a ceramic heater. (b) Compressive force–strain curves of HCCs with different hydrogel-coating layers. The insert shows the substantial deformation of an HCC without bursting upon compression. (c) The maximum compressive force as a function of the number of coating layers for HCCs swelled in SGF and NS. Data are presented as mean ± SD; *n* = 3 independent experiments. (d) Optical images showing the process of compression and recovery for a 4-HCC with 60% solid content hydrogel coatings. (e) Cyclic compressibility of a 4-HCC with 60% solid content hydrogel coatings, with the compression deformation rate controlled at 40%. (f) Heat map showing the impact resistance and the resilience of HCCs with different numbers of 60% solid content hydrogel-coating layers. Cross and circular symbols represent the occurrence and absence of failure, respectively. (g) Keyframes extracted from high-speed photography capturing a 4-HCC falling from a height of 40 cm (results corresponding to the point selected by a black frame in (f)). (h) Comparison of residual properties between MLMs and HCCs on porcine skin tissues.

HCCs are reliable to resist large deformation and impact. We firstly detected the deformation capability of an HCC with 60% solid content hydrogel coating by using compression tests. The temperature was consistently maintained at ∼50°C during the tests to ensure that the inner liquid metal remained in a liquid state (Fig. 3a). These HCCs could withstand compressive strains of >85% without structural failure (Fig. 3b). The maximum compressive force increased with the number of hydrogel-coating layers, where the maximum compressive force of a 5-HCC was >65 N. We also measured the compressive reliability of HCCs after immersion in SGF and NS (Fig. 3c and Fig. S20). As the modulus of the hydrogel coating decreased by swelling, the maximum compressive forces for swelled HCCs ranged between 0.3 and 6 N, translating to compressive strengths of 2.7–53.1 kPa. The compressive strength of 4-HCC (∼39.9 kPa) and 5-HCC (∼46.3 kPa) surpassed the maximum gastric pressure in humans, which ranges from 5 to 13 kPa [54]. We should note that a constantly peristaltic gastrointestinal tract can periodically compress swallowed devices (∼1000 cycles per day in the stomach [55]). Thus, we conducted the cyclic compression tests on the 4-HCCs with a 60% solid content hydrogel coating. The 4-HCC rapidly recovered to the initial spherical shape after undergoing 85% compressive strains (Fig. 3d, Fig. S21 and Movie S4). It also resisted >2000 compression cycles without fatigue failure (Fig. 3e). Moreover, cyclic compression tests for 4-HCCs swelled in SGF implied that the capsules were capable of withstanding the stomach peristalsis (Fig. S22).

We evaluated the impact resistance of HCCs by using a drop test, in which HCCs were dropped from predetermined heights onto a plastic board (Movie S5). The maximum drop height was set at 40 cm to approximate the length of the human esophagus. The impact resistance of the HCC increased with the number of hydrogel-coating layers (Fig. 3f and Fig. S23). 1-HCCs and 2-HCCs ruptured upon falling from heights of 8 and 24 cm, respectively (Fig. S24a). When more than three coating layers were applied, the HCCs could endure a substantial impact, meeting the requirements for safe use. For example, a 4-HCC could withstand the strong impact of a fall from a height of 14 m (Fig. S24b and c). Additionally, the HCCs showed excellent elasticity, bouncing back after impact. The elasticity of the HCCs also increased with the number of coating layers. Here, a 4-HCC could rebound multiple times after falling from a 40-cm drop, reaching a first bounce height of 22.1 mm. No leakage of MLM was detected after the HCCs fell and bounced (Fig. 3g). Furthermore, in comparison with the metal residues and spatters observed when a MLM droplet was dropped onto fresh porcine skin, the HCC remained intact and could be easily removed without any residue (Fig. 3h). This significant advantage also existed when the substrates varied (Fig. S25). Based on the above results, we chose 4-HCCs as candidates for subsequent applications.

### On-demand magnetic manipulation

**Figure 4.**
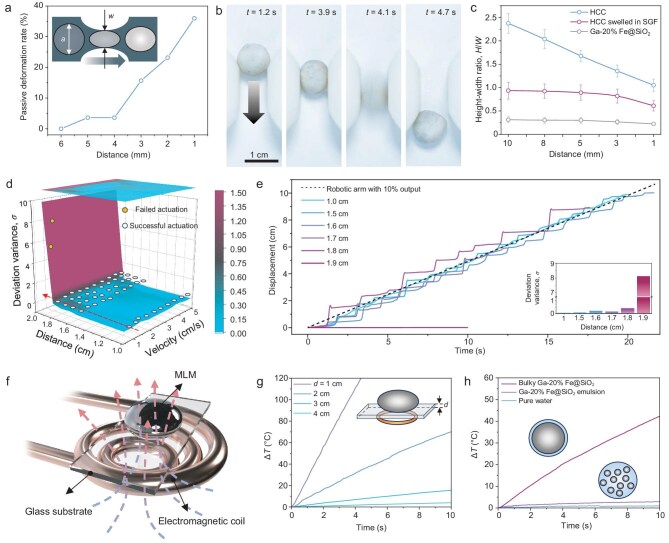
Deformation, locomotion and wireless heating of 4-HCC in magnetic fields. (a) Passive deformation of HCCs through narrow channels. The passive deformation rate increases as the magnet approaches the sample. The inset illustrates the set-up for the passive deformation test. *a* is the diameter of the HCC, *w* is the width of a narrow channel and 1-*w*/*a* represents the passive deformation ratio. (b) Keyframes showing the passive deformation process of HCCs to pass through narrow channels. (c) Changes in the geometry of HCC, HCC swelled in SGF and MLM (Ga-20% Fe@SiO_2_) as the magnet–sample distances decrease. (d) Motion synchronization between HCC and a moving magnet fixed on a robotic arm for actuation, at various actuation distances and under different actuating velocities. (e) Locomotion dynamics of HCCs at a constant speed of 0.5 cm/s from various distances (red dotted line in (d)). The insert diagram shows the corresponding deviation variances. (f) Schematic illustrating the set-up for characterizing the magnetic induction heating of MLM on a glass sheet. (g) Temperature evolution of MLMs by magnetic induction heating when an alternating magnetic field is applied from different distances. The inset depicts the definition of the distance *d*. (h) Temperature evolution of bulky Ga-20% Fe@SiO_2_, Ga-20% Fe@SiO_2_ emulsion and pure water under an alternating magnetic field, where all coil–sample distances are controlled at 2 cm.

HCCs can achieve controllable deformation in magnetic fields. We first measured the passive deformation behaviors of HCCs under a magnetic field. HCCs could passively adapt to their surroundings, enabling them to navigate through narrow channels under magnetic actuation (Fig. 4a and b, and Movie S6). This passive deformation capability of HCCs could be enhanced by decreasing the magnet–HCC distance (a maximum passive deformation rate was measured at ∼36.0% when the distance was 1 mm). In contrast, while an MLM possessed a higher passive deformation rate, it also produced massive metal residues on the substrate (Fig. S26b). Meanwhile, a magnetic silicone ball generated no by-product residues but struggled to pass through channels due to poor passive deformability (Fig. S26c). These results highlighted the superior adaptability and cleanliness of HCCs in applications that require deformation and movement in confined spaces. Then, we measured the active deformation behaviors of HCCs under a magnetic field (Fig. 4c) and compared them with swelled HCCs and MLMs (Fig. S27). When the magnetic flux density increased by bringing a magnet close to the HCC (Fig. S28), it deformed actively and led to a decrease in the height–width ratio (*H*/*W*) following the magnetowetting dynamics [56]. The inherent elasticity of HCCs allowed a better linear correlation between the active deformation and the controlled magnet–sample distance, as demonstrated by the compared responses of swelled HCCs and MLMs.

HCCs can achieve governable movement in magnetic fields. This capability was demonstrated by using a robotic arm fixed with cylindrical permanent magnets (N52, 15 mm in diameter × 20 mm in height) to actuate HCCs on wet porcine skin at varying velocities and magnet–HCC distances (Fig. S29 and Movie S7). A deviation variance $\sigma = \frac{{\mathop \sum \nolimits_{i = 1}^n {{( {{x_c} - {x_r}} )}^2}}}{m}$ was introduced to quantify the synchronization between the magnet and the HCC, where *m* is the number of sampling points, and *x*_c_ and *x*_r_ are the instantaneous displacement of the capsule and the robotic arm, respectively. A larger *σ* represents poor synchrony between the movements of the magnet and the HCC (Fig. 4d). The results showed that the magnet was able to actuate HCCs at a speed of 5 cm/s when the magnet–HCC distance was <1.8 cm. The displacement–time curves of the magnet closely overlapped that of the HCC, with *σ* being <0.15 for distances of <1.6 cm, indicating excellent synchronization (Fig. 4e). However, at greater distances, the synchronization largely worsened due to the unavoidable rolling friction that was encountered on the rough surface of the porcine skin. Attempts to actuate the HCC from distances of >1.9 cm were unsuccessful. Moreover, inappropriate actuation velocities resulted in poor synchronization (Fig. S30). HCCs tended to stick to the substrate at low speed and the motion inertia was large at a high speed, yielding a high *σ.* The optimal synchronicities (*σ* < 0.15) occurred at an actuation speed that ranged from 2 to 3 cm/s. Based on the governable movement capabilities, HCCs could be manipulated to trace trajectories of ‘Z’, ‘J’ and ‘U’ letters on demand under the actuation of the magnet (Movie S8). It is worth noting that a higher concentration of inner magnetic particles in HCCs enables a stronger magnetic response for the desired governable movement (Fig. S31).

### Remotely targeted thermal ablation using HCCs

HCCs can achieve wireless heating when subjected to high-frequency alternating magnetic fields (AMFs) due to the inherent magnetism and conductivity of MLMs [12]. A water-cooled electromagnetic coil connected to induction heating equipment was used to generate AMFs for heating (Fig. 4f and Fig. S32). Exposed MLMs were selected to represent HCCs during heating to facilitate more accurate detection of temperature changes. We found that the inclusion of Fe@SiO_2_ in MLMs improved their heating efficiency (Fig. S33a). Moreover, the coil–MLM distances dictated the heating efficiency (Fig. 4g). When this distance was <3 cm, the achieved heating efficiency met the levels required for traditional thermal ablation therapy [57]. The heating efficiency of bulk MLMs was significantly higher than that of Ga-20% Fe@SiO_2_ emulsion (appearance in Fig. S33b) and pure water (Fig. 4h). This superiority in heating efficiency is attributed to the bulk formation of MLMs within the containment capsule, highlighting the potential of these capsules in applications that are related to remote thermal therapy.

**Figure 5.**
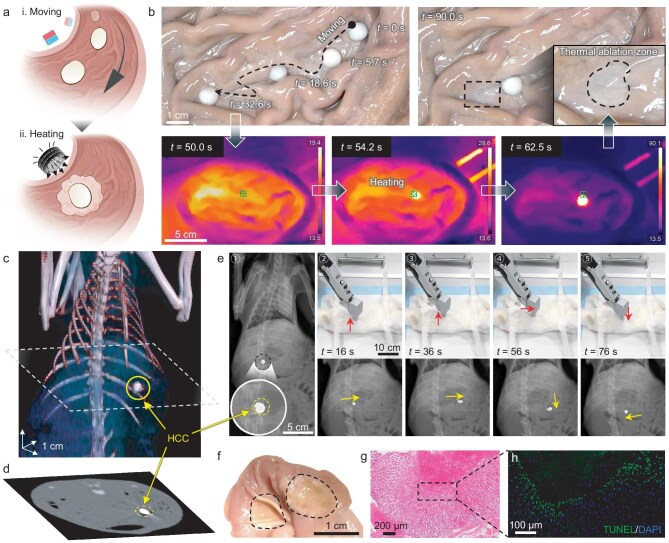
*Ex vivo* and *in vivo* remotely targeted thermal ablation operations using HCCs. (a) Schematic of remotely targeted thermal ablation using HCCs under the actuation of a magnet and wireless heating by an alternating magnetic field. (b) Experimental demonstration of targeted thermal ablation in an *ex vivo* porcine stomach using HCCs. The curve with an arrow represents the motion trail of the HCC and the zoomed-in image shows the details of scalded tissue. The maximum temperature reaches 90.1°C within ∼10 s of heating. (c) Visualization of the 3D HCC by using abdominal computed tomography combined with a volume-rendering technique. (d) Cross-section image selected from (c) locating the position of the swallowed HCC. (e) Sequential X-ray images and corresponding snapshots showing magnetic manipulation of HCC in a rabbit stomach model. The red arrows represent the motion direction. (f) Optical image showing scalded gastric tissues from an *in vivo* rabbit model. (g) Photomicrographs of hematoxylin and eosin staining in the paraffin-embedded scalded gastric tissue from rabbits after thermal ablation operation, where numerous red blood cells effuse in the gastric mucosal layer. (h) Terminal-deoxynucleotidyl transferase mediated nick end labeling (TUNEL) staining from rabbits after thermal ablation operation. Channels are: 4′,6-diamidino-2-phenylindole (DAPI) nuclear stain (blue) and apoptosis by TUNEL (green). Image (a) created with BioRender.com, with permission.

In previous studies, biological tissues are in the transparency to low-frequency magnetic field (0–400 kHz) [11,58,59] so that the attenuation effect of the magnetic field in the body to the operations of the capsule is negligible. Consequently, we demonstrated the use of HCCs for remotely targeted thermal ablation in an *ex vivo* porcine stomach model. This operation consisted of two steps (Fig. 5a). Initially, an HCC was actuated to the desired location by using a static magnetic field. Subsequently, an AMF was applied to rapidly induce heating for the thermal ablation procedure. Throughout the therapy, the hydrogel coating of the HCC maintained its spherical shape, enhancing its ability to roll and navigate across substantial gastric folds. This enabled the HCC to reach the targeted position in ∼30 s. An electromagnetic coil was then positioned ∼2 cm away from the HCC and the AMF was activated for wireless heating. As a result, the temperature of the HCC was raised from 17.7°C to 89.4°C in ∼10 s. This temperature was high enough for hyperthermia destruction of the targeted tissue, creating a thermal ablation zone (Fig. 5b and Movie S9).

### *In vivo* demonstration in rabbit digestive tract model

A fully soft HCC system is capable of medical image tracking, remote motion control and the active performance of thermal ablation compared with traditional tethered endoscopy and rigid capsule robots [60–62]. Therefore, *in vivo* experiments were conducted to verify the feasibility of HCCs for theragnostic gastrointestinal operation. We first evaluated the biocompatibility of HCCs. The results of cell viability and Live/Dead assays showed that the leach liquors from HCCs exhibited low cytotoxicity towards various normal cell lines including NCM460 (normal human colon mucosal epithelial cell), GES-1 (human gastric epithelial cell) and 293T (human embryonic kidney cell) (details in Fig. S34). A proof-of-concept study involving oral administration was demonstrated in rabbit models. Selective HCCs, with their suitable size (6–8 mm in diameter) and flexibility, were easily ingested by unanesthetized rabbits (Fig. S35a). Subsequently, the ingurgitation of the HCC was confirmed by using X-ray imaging (Fig. S35b). We note that the HCC is highly detectable by X-ray due to the higher density of the MLM compared with the surrounding biological tissues. This property facilitated the tracking of HCCs during *in vivo* operations. The swallowed HCC successfully passed through the gullet due to its satisfactory passive deformability and reached the stomach without rupture even after enduring the compression of esophageal peristalsis. The 3D reconstruction image confirmed the location of the HCC and structural integrity in the stomach (Fig. 5c and d). After residing in the moist and acidic gastric environment for >4 hours, the hydrogel coating of the HCC still maintained its mechanical integrity, effectively preventing the leakage of inner MLMs despite the constant motion and pressure from gastric peristalsis. In contrast, swallowed MLM droplets without containment scattered, leaving metal residues in the rabbit oral cavity and stomach (Fig. S36).

To avoid radiation risks during operation, a robotic arm equipped with a spherical permanent magnet (N48, 25 mm in diameter) was programmed to govern the locomotion of the swallowed HCC (Fig. 5e and Fig. S37). The HCC was able to respond to the external magnetic field and moved smoothly along the predetermined trajectory (Movie S10). No leakage or adhesion of liquid metal inside the stomach during motion was observed. Upon reaching the targeted position, the electromagnetic coil was deployed to produce AMFs, enabling wireless heating of the HCC (Fig. S38). The heating process resulted in notable damage to the targeted gastric mucosa surface and peripheral tissues (Fig. 5f and Fig. S39), as evidenced by the disrupted arrangement of glandular structures and the presence of hemorrhagic and erosive lesions (Fig. 5g and Fig. S40). The results of immunofluorescence staining in the tissue from rabbits after the remote thermal ablation operation proved that the hyperthermal HCC caused effective damage to the stomach tissue (Fig. 5h and Fig. S41). Subsequent anatomical examination further verified the structural integrity of the HCC after ingestion and magnetic manipulation (Fig. S42).

## CONCLUSION

In summary, we fabricated a tough HCC of MLM by using an effective coating technique that was inspired by the craft of making rice glue balls. This approach resulted in formed hydrogel coatings with outstanding mechanical performances, including high stretchability (∼500% elongation), satisfactory fracture energy (3362.1 J/m^2^ during long-term storage and 262.9 J/m^2^ after swelling) and strong interfacial bonding to liquid metal (425.3 J/m^2^). As a result, the obtained HCCs have remarkable mechanical reliability, capable of withstanding violent compression strains of ≤85% and impacts from drops of >14 m. These HCCs also show long-term stability to withstand cyclic compression for ≤2000 cycles. The simple approach to fabricating a tough hydrogel coating on the surface of liquid metal is suitable for mass production, opening up several possibilities for future research and applications. For example, the hydrogel interface that forms between the liquid metal and its external environment facilitates different physicochemical phenomena. This interface shows promise as a channel for the selective transmission of specific substances between the encapsulated liquid metal and the external environment. The soft containment strategy also retains the original softness of the liquid metal as much as possible for flexible storage and transportation. Importantly, the encapsulation of bulky liquid metals prevents their dispersion and adhesion to biological tissues. It addresses the intractable residue issue of liquid metal, paving the path for future safe medical applications. Meanwhile, the capabilities of HCCs for governable movement and wireless thermal ablation in theragnostic gastrointestinal operation are demonstrated. Wireless thermal ablation using HCCs shows promise in eliminating intestinal metaplasia in Barrett's esophagus and mitigating the recurrence of gastrointestinal polyps [63,64]. This containment hydrogel capsule system also holds potential for applications in targeted drug delivery, molecular imagery and implantable medical devices [65].

## ETHICAL STATEMENTS

This work was performed in accordance with the recommendations in the *Guide for the Care and Use of Laboratory Animals* and relevant Chinese laws and regulations. All the animal assays were approved by Institutional Animal Care and Use Committee of Zhejiang University (approval number: ZJU20230523) and postoperative care was supervised by the staff in the Animal Experimental Center of Sir Run-Run Shaw Hospital, Zhejiang University.

## Supplementary Material

nwaf042_Supplemental_Files
