# Supplementary material for: Tough hydrogel-coated containment capsule of magnetic liquid metal for remote gastrointestinal operation
Source: Natl Sci Rev. 2025 Feb 11;12(4):nwaf042. doi: 10.1093/nsr/nwaf042 (PMC11892562; doi:10.1093/nsr/nwaf042)
Supplement: nwaf042_Supplemental_Files [file nwaf042_supplemental_files.zip › Supplementary data.pdf]

Supplementary Information

Tough Hydrogel-Coated Containment Capsule of Magnetic Liquid Metal for Remote Gastrointestinal Operation

Yifeng Shen[1,2#], Jiasheng Cao[3,4#], Enjie Zhou[3,4#], Lei Wang[1,2], Kaihang Zhang[1,2], Yaoting Xue[1,2], Hui Yuan[1,2], Jiahao Hu[3,4], Siyang Li[1,2], Zhikun Miao[1,2], Yukai Zhao[1,2], Tuck-Whye Wong[5], Tiefeng Li[1,2*], Mingyu Chen[3,4*], Xuxu Yang[1,2*] and Wei Yang[1,2]

[1]Center for X-Mechanics, Department of Engineering Mechanics, Zhejiang University, Hangzhou 310027, China;
[2]Department of Engineering Mechanics, Zhejiang University, Hangzhou 310027, China;
[3]Department of General Surgery, Sir Run-Run Shaw Hospital, Zhejiang University, Hangzhou 310016, China;
[4]Zhejiang University School of Medicine, Zhejiang University, Hangzhou 310058, China;
[5]School of Biomedical Engineering and Health Sciences and Advanced Membrane Technology Research Centre, Universiti Teknologi Malaysia, Skudai 81310, Malaysia

*Corresponding authors. E-mails: litiefeng@zju.edu.cn; mychen@zju.edu.cn; xxyang@zju.edu.cn
[#]Equally contributed to this work.

This PDF file includes:

Supplementary Movie 1 to 10

Experimental methods

Materials and characterization

Material preparation

Material characterization and mechanical property measurement

Experimental details of *ex vivo* and *in vivo* demonstrations

Supplementary Table 1 to 3

Supplementary Figure 1 to 42

References

Supplementary Movie

Supplementary Movie. 1

Self-gelling process of lyophilized hydrogel powder in the glycerol aqueous solution.

Supplementary Movie. 2

Tensile tests for hydrated hydrogels with 60% solid content with and without a notch.

Supplementary Movie. 3

90°-tearing test for self-gelling hydrogel coatings on a solidified magnetic liquid metal substrate.

Supplementary Movie. 4

Compression process and recovery for 4-HCC.

Supplementary Movie. 5

Impact resistance for HCCs with different hydrogel layers when falling from different heights.

Supplementary Movie. 6

Passive deformation of HCC in a narrow channel under the actuation of magnet.

Supplementary Movie. 7

The motions of HCC on fresh porcine skins under the actuation of a magnet with different velocities and distances.

Supplementary Movie. 8

Writing “Z”, “J”, and “U” letters to show on-demand movement for HCC under the actuation of a magnet.

Supplementary Movie. 9

Targeted thermal ablation using HCC in *ex vivo* porcine stomach.

Supplementary Movie. 10

Magnetic manipulation for HCC in *in vivo* rabbit stomach models under the actuation of magnet fixed on robotic arm.

Experimental methods

Materials and characterization

In the process of fabrication, iron particles (Leber, 99.9%), tetraethylortho silicate (TEOS, Sigma-Aldrich, 98%), pure gallium (Dongguan Dingguan Metal Technology Co., Ltd, 99.999%), acrylic acid (AAc, Aladdin, 99%), 3-(trimethoxysilyl)propyl methacrylate (TMSPMA, Sigma-Aldrich, 98%), α-Ketoglutaric acid (α-keto, Sigma-Aldrich, 99%), N,N′-methylenebisacrylamide (MBAA, Sigma-Aldrich, 99.5%), glycerol (Macklin, 99%), ammoia solution (Aladdin, 25-28%), polyvinylpyrrolidone (PVP, Aladdin, K30), simulated gastric fluid (SGF, pH = 3, Dongguan Chuangfeng Automation Technology Co., LTD), sodium chloride (NaCl, Sinopharm Chemical Reagent Co., Ltd, AR) were commercially purchased and used as received. Porcine stomachs and skins were purchased from a local fresh food market. Cylindrical permanent magnets (N52, 15 mm in diameter × 5 mm in height), cube magnet (N52, 10 mm × 10 mm × 10 mm) and spherical permanent magnets (N48, 25 mm in diameter) were purchased from Shenzhen Lala Magnetic Material Development Co., Ltd. Copper sheets, glass sheets, acrylic sheets were purchased from Taobao. In the process to evaluate the cell viability of HCCs, Dulbecco’s modified eagle medium (DMEM, Gibco), fetal bovine serum (Cellmax), 0.25% trypsin (Solarbio), phosphate buffer saline (Sigma-Aldrich), penicillin streptomycin combination (Gibco), Live/Dead viability/cytotoxicity kit (L3224, Thermo Fisher Scientific), and Cell Counting Kit 8 (CCK8, Abcam, ab228554) were commercially purchased and used as received. During the process of *in vivo* experiment and biological tissue analysis, pentobarbital sodium (60 mg/kg, i.v., Sigma-Aldrich), 4% paraformaldehyde (Beyotime), dimethylbenzene (Sigma-Aldrich), and hematoxylin-eosin (Sigma-Aldrich) were commercially purchased and used as received.

The lyophilized hydrogel powders (LHPs) were prepared by a freeze dryer (Shanghai Lichen Instrument Technology Co., LTD, LC-18N-50B). The FT-IR spectra of LHPs was obtained by Fourier-transformed infrared spectrometer (Nicolet380, Thermo Fisher Scientific). The microstructures of LHPs, interfaces between hydrogel coating and magnetic liquid metal (MLM), and Fe@SiO_2 magnetic particles were observed by a scanning electron microscope (ZEISS, Sigma 300). The magnetic hysteresis loops of various MLMs were measured by a vibrating sample magnetometer (LakeShore7404). The size distribution of LHPs was obtained by a laser particle analyzer (Malvern Mastersizer 2000). The XRD diffraction patterns were obtained by X-ray diffractometer (Rigaku SmartLab SE). The micro-CT analysis was characterized by three-dimensional X-ray microscopy (Zeiss Versa 515). The self-gelling process of LHPs in solutions was recorded by an optical microscope (PLYMPUS). The evolution for rheological performance of LHPs in solutions were measured by a rheometer (Anton Paar, MCR302). The rheological performance of hydrated hydrogel with different glycerol contents were measured by another rheometer (Haake Mars40). The dropping and bouncing process of hydrogel-coated containment capsule (HCCs) of magnetic liquid metal was observed by a high-speed camera (Chronos cr2.1). Resinous holders crafted via photopolymerization 3D printing (Formlabs, Form 3+). The mechanical performances of hydrogel and HCC were measured by a static material testing machine (ZwickRoell, ProLine Z10). A laser engraving machine (Trotec) was used to cut

polyimide films. The electrostatic spraying was achieved by an electrostatic spraying machine (MS-K19). Magnetic liquid metal emulsions were prepared by a probetype sonicator (JY92, Ningbo Xinzhi Biotechnology Co., Ltd) at 60% power for 3min. The strength of magnetic field was measured by a gaussmeter (Sanliang, TS100). The wireless heating for thermal ablation operation was achieved by a high-frequency induction heating equipment (Ningbo Guosheng Electronic Equipment Co., LTD, GHG-10KW). The temperature changes upon heating were recorded by a temperature sensor (Daga Sensor) and a handheld thermal imager (HIKMICRO H13). Laser confocal microscope (Leica) and Multiskan GO (Thermo Fisher Scientific) was used to evaluate the cell viability. A dual-source 128-slice CT scanner (Siemens SOMATOM Definition AS, Erlangen, Germany) and a fluorospot X-ray image system (Siemens Fluorospot Compact FD, Erlangen, Germany) were applied for medical imaging. A Zeiss fluorescence microscope (Carl Zeiss GmbH, Oberkochen, Germany) was used to capture the fluorescence signals of the images.

Material preparation

Preparation of hydrogel-coated containment capsule (HCC)

1) Preparation of lyophilized hydrogel powder (LHP)

To fabricate the pre-polymer mixture, one starts with combining 160 mL of distilled water (solvent), 80 mL of acrylic acid (monomer), 2.1 mL of 0.1 M MBAA (crosslinker), 600 μL of 0.1 M α-keto (initiator), and 120 μL of TMSPMA. Stir this mixture vigorously for about 3 minutes to ensure the TMSPMA, which initially exhibits hydrophobic properties, is evenly dispersed. The addition of α-keto creates an acidic environment that aids in the hydrolysis of the silane groups but prevents their condensation. Following this, the mixture is transferred into 50 mL syringes, from which any air bubbles are removed using ultrasonication. The syringes are then sealed in an environment filled with nitrogen gas. The next step involves curing the mixture under UV light (365 nm, 500 mJ/cm^2 power) for 5 hours, keeping a constant distance of 5 cm between the light source and the samples to ensure uniform exposure. After curing, the gels are carefully extracted from the syringes and placed in distilled water to leach out any unreacted monomers and other residual chemicals. The water needs to be changed every 24 hours, with a total of 3 changes required to ensure thorough purification. Once cleaned, the obtained p(AAc-*co*-TMSPMA) hydrogels are frozen in liquid nitrogen, ground into smaller pieces, and then freeze-dried for 48 hours. The final product, a finely powdered hydrogel, is sifted through a 100-mesh screen to ensure uniformity and stored in a desiccator for preservation and future use.

2) Preparation of solidified MLM ball

Before mixing with gallium, iron microparticles should be modified by inert silica layer to attenuate reactions between organic acid and active iron powder (Fig. S3). Therefore, a solution consisting of 400 mL of anhydrous ethanol, 100 mL of distilled water, and 5 mL of ammonia solution is prepared in advance. Then, 20 g of spherical iron powder are dispersed in the above solution under ultrasonication for about 5 minutes. During vigorous mechanical stirring at 300 rpm, 6 g of TEOS is slowly added dropwise for controlling reaction speed. After 24 hours of stirring, a magnet is used to separate the obtained magnetic powders and wash them with ethanol for 3 times. These powders are dried in a vacuum oven at 60 ℃. Then, we mix 4 g of Fe@SiO_2 powders with 16 g of pure gallium in a mortar for about 2 minutes, thus obtaining Ga-20wt% Fe@SiO_2 composites. Other magnetic liquid metals (MLMs) with different magnetic particle mass ratios (10wt%, 30wt% and 40wt%) can be prepared by the same method. Finally, the composite pastes are transferred into spherical molds using a pipette and is frozen overnight at -20 ℃ to obtain solidified MLM balls.

3) Rolling and coating to prepare HCC

At room temperature (25°C), a 20% glycerol aqueous solution sprays on the surface of the solidified MLM ball, where the glycerol water droplets wet on the surface of MLM ball due to the hydrophilic nature of the liquid metal. Then, the MLM ball rolls in a dish filled with lyophilized gel powder. The porous LHPs swell rapidly upon contact with water on the metal surface, exposing numerous carboxyl groups and uncondensed silanol groups on the polymer chains. Then, the powder adheres effectively to the surface of MLM ball under the influences of hydrogen bond and Si-O-Si covalent bond. This covalent bond is formed from dehydration condensation between the silicon hydroxyl groups of TMSPMA in the polymer chains and the gallium hydroxide groups on

the surface gallium oxide layer. During the rolling process, loosely adhered powders on the surface can be shaken off, preventing excessive accumulation in specific surface areas. The rapid mechanical rolling process compresses the powders under impact, resulting in a smoother surface. Finally, the 20% glycerol solution sprays again to fully swell and hydrates the LHPs on the surface of MLM ball. It enables the self-gelation among LHPs to form a complete micron-thick hydrogel coating on the MLM ball. The process can be repeated as needed to construct HCCs with a controllable coating thickness, where HCCs with one hydrogel layer (1-HCC), two layers (2-HCC), three layers (3-HCC), four layers (4-HCC) and five layers (5-HCC) are prepared in our paper.

Preparation of tensile specimens and compression specimens fabricated by hydrated LHPs

Initially, 2 g of LHPs and 8 g of pure water solution (SGF or NS) are mixed and thoroughly stirred for 20~30 minutes to ensure dispersion of moisture throughout the dry powder. Upon absorbing water, LHPs rapidly swell and adhere together to form a bulky hydrogel. This hydrogel with an irregular shape is evenly divided into four portions. Each part is molded into a long strip in polytetrafluoroethylene (PTFE) molds (80mm × 20mm × 3mm), where the shape is suitable for subsequent tensile testing. Then, another PTFE board is used to cover the molds, and dovetail clips are applied to firmly encase the hydrogel to prevent the water loss. Subsequently, the entire assembly is placed in a sealed bag containing water-saturated paper to prevent excessive dehydration, and the assembly is then kept in an oven at 60℃ for 24 hours (or in normal environment for 2 days). The moisture content of obtained hydrogel can be close to 80% (~20% solid content). To achieve a hydrogel with ~40% moisture content (~60% solid content), the high-moisture hydrogel undergoes further drying in an oven at 60℃ for approximately 1 hour. Finally, we prepare tensile specimens with different solid contents fabricated by hydrated LHPs.

The preparation process for compression specimens is similar to that for tensile specimens. 1 g of LHPs and 4 g of pure water (1 g of LHPs and 0.7 g of pure water for hydrogels with 60% solid content) are mixed and thoroughly stirred for 20~30 minutes to ensure dispersion of moisture throughout the dry powder. Then, the hydrogel samples are cut into same cylindrical shape (a height of 10 mm and a diameter of 8 mm) for standard compressive tests.

Preparation of Ga-Fe@SiO_2 emulsion

After mixing with Fe@SiO_2 and gallium, 10g of obtained Ga-20% Fe@SiO_2 is immersed in 10ml of 10wt% polyvinyl pyrrolidone (PVP) ethanol solution. The emulsion is prepared using an ultrasonic probe (60% power for 3min). The appearance of the fabricated emulsion is shown in Fig. S33b.

Material characterization and mechanical property measurement

Interface characterization for HCC

4-HCCs are placed in an oven at 80 ℃ for one day to dry surface hydrogel coatings for the ease of observation. These dried 4-HCCs are transferred to a sealed bag and placed in a -20 ℃ freezer to solidify the inner MLM core. Some 4-HCCs are also treated by lyophilization for one day. Two kinds of surface-dried and internally solidified 4-HCCs are broken by means of a hammer, and the interfaces of selected pieces are observed by camera for dried samples and scanning electron microscope (SEM) for lyophilized samples.

Rheological property of LHP during hydration

An adequate amount of LHPs (over 100 mg) fills the space in the parallel plates of a rheometer, maintaining a gap height of 1mm. The equipment is set to an oscillation mode for modulus testing, and tests are conducted at a fixed amplitude of 0.1% and a fixed frequency of 1 Hz. Once the test data stabilize, a small amount of pure water (about 1 mL) is introduced from the side of the parallel plates, activating the hydration for LHPs. The changes in the rheological properties are recorded during the process.

Rheological performance of hydrogels with various glycerol contents

0.5 g of LHPs and 2 g of aqueous solutions with different glycerol contents (0%, 20%, and 40%) are mixed and thoroughly stir for 20~30 minutes to ensure dispersion of moisture throughout the dry powder. Then, the obtained hydrogels are pressed into a round disc shape with approximately uniform dimensions (a thickness of ~2 mm, a diameter of ~3.5 mm). The rheometer is set to an oscillation mode for modulus testing with varying frequencies, and tests are conducted at a fixed amplitude of 0.1%.

The strength of magnetic field may change the rheological behavior of magnetic liquid metal[1,2]. However, the magnetic liquid metal is encapsulated within hydrogel coatings, which exhibit minimal response to magnetic fields. As a result, the overall rheological changes of the HCC system are complex and warrant further investigation in future studies.

Tests for mechanical properties

Tensile test

Tensile specimens of hydrated hydrogel with 40% water content, 80% SGF content, 80% NS content, and 80% water content are clamped in a tensile machine at room temperature and constant humidity (~25 ℃, ~60% RH), and then are pulled at a speed of 150 mm/min.

Fracture toughness test

Unnotched and notched tensile samples of hydrated hydrogel with 40% and 80% water contents are clamped in a tailor-made fixture (Fig. S15) at room temperature and constant humidity (~25 ℃, ~60% RH). The samples are pulled at a speed of 15 mm/min. The test process is shown in Movie. S2.

Lap shear test

Firstly, Ga-20wt% Fe@SiO_2 composites are brushed onto glass slides to form thin MLM films. The mixing process leads to the generation of a large number of oxides in the MLM composites,

allowing the composite paste to be brushed to the substrate easily and uniformly. The prepared substrates are refrigerated overnight at -20 ℃. Before electrostatic spraying for LHPs, an acrylic piece is used to cover areas not desired for powder coating, and to expose a 2 cm × 2.5 cm area for subsequent coating and adhesion. During electrostatic spraying, a grounding electrode is connected to the solidified MLM film, and the pre-prepared LHPs are sprayed using an electrostatic spray gun (80 kV, 60 μA). The powders adhered to the MLM film are hydrated using a spray gun loaded with 20% glycerol aqueous solution. Another glass slide without powder but with MLM layer, is pressed onto the sticky hydrated LHP layer (~2 kPa pressure for 10 minutes). After 12 hours, the samples are clamped on both sides by a tensile machine and pulled at a speed of 20 mm/min at room temperature and constant humidity (~25 ℃, ~60% RH). The test demonstration is shown in Fig. S18.

90 °-tearing test

The Ga-20wt% Fe@SiO_2 composites are brushed onto glass slides, and the prepared substrates are refrigerated overnight at -20 ℃. Polyimide films (PI film, 3 μm-thickness) are cut into 70 mm × 8 mm strips by laser. Using tape, one adheres one end of the PI film to one side of solidified MLM layer. The LHP layers are uniformly constructed on the surface using electrostatic spraying same as above methods, followed by hydrating with 20% glycerol aqueous solution. After 24 hours, one free end of PI film is clamped by the tensile machine and pulled in a direction perpendicular to the MLM substrate to tear the covered hydrogel coating. The test demonstration is shown in Fig. S19 and Movie. S3.

Compression test

Compression tests are performed to measure the resistance to compression of HCCs with different numbers of hydrogel coating layers. An Al_2O_3 ceramic heater (3.7 V, 2 W) is placed below HCCs to avoid inner liquid metals solidification. Firstly, HCCs with 60% solid content hydrogel coatings are compressed in a test machine. Then, we immerse HCCs in SGF and NS for 24 h to a swelling equilibrium state. These swelled HCCs with different numbers of hydrogel coating layers are compressed under the same test conditions. Additionally, the cyclic compressibility of 4-HCC with 60% solid content hydrogel coating and swelled 4-HCC are measured by the test machine, where the extent of compression is controlled to 40%.

Finite element simulation of compression for HCC

All simulations are carried out using ABAQUS 2020. The ABAQUS/Explicit solver is employed for the simulations. Models of the hydrogel coating are constructed using 4-node element with bilinear axisymmetric quadrilateral, reduced integration, hourglass control (ABAQUS element type CAX4R). The hydrogel material behavior is described as hyperelasticity with 3rd strain energy potential under Ogden model fitting from the uniaxial tension test data. The hydrogel coating is modeled as fluid cavity with inner liquid metal of 6 g/cm^3 density. The diameter of HCC is designed to be 8mm, and the hydrogel coating thickness is designed as 500 μm. The liquid metal-filled hydrogel capsule is placed between two rigid plates. The compression test process is conducted with one rigid plate moving toward the other.

Deformation, locomotion, and induction heating performance for HCC under magnet fields

Magnetic responsiveness

We choose 4-HCCs with different magnetic particle contents (5%, 10% and 20%) and a permanent magnet (N52, 10 mm × 10 mm × 10 mm) to perform experiments (as shown in Fig. S31). A piece of glass slide (1 mm thickness) acts as the experimental track. The magnet is positioned directly beneath the glass, while the 4-HCC is placed on top of the glass. The magnetic 4-HCC will roll towards the magnet under the influence of magnetic field gradient. The maximum distance to be driven refers to the distance between the location of the magnet and the point where the 4-HCCs begin to move. The v and a represent the velocity and accelerated velocity of 4-HCC during magnetic actuation, respectively.

Active deformation performance

Samples such as 4-HCC, swelled 4-HCC, and uncoated Ga-20wt% Fe@SiO_2 are tested. Each sample is placed above a cylindrical permanent magnet (N52, 15 mm in diameter × 5 mm in height). A number of stacked glass slides (1 mm thickness every slide) are used to control the distance between the sample and the permanent magnet. Under the influence of a magnet, the active changes in the height and width of samples are recorded and evaluated.

Passive deformation performance

The tests select 4-HCCs for evaluation. 4-HCCs (diameter is a) are placed into 3D printing channels (made by Shenzhen Wenext Technology Co., Ltd.) with different slit widths (width is w). A cylindrical permanent magnet (N52, 15 mm in diameter × 5 mm in height) is placed directly beneath 4-HCCs to drive them to pass through the channels. The narrowest width through which a 4-HCC can pass is used to demonstrate its passive deformation capability, where the passive deformation ratio is calculated by $1\text{-}w/a$. The passive deformation ratio of 4-HCCs under different magnetic field strengths is tested under different distance between the capsule and the permanent magnet. A small amount of glycerol aqueous solution is sprayed into the channels to reduce friction between the capsule and the channel inwall. Uncoated MLMs (Ga-20wt% Fe@SiO_2) and magnetic silicone balls (0.4 g iron powders mixed within 1.6 g polydimethylsiloxane elastomers) are also tested for comparison. The same permanent magnet is used to drive the uncoated MLM and the magnetic silicone ball through a channel when keeping the distance between the magnet and the MLM at 1 mm. The processes of passing through channel for 4-HCC, MLM and magnetic silicone ball are shown in Fig. S26.

Movement performance under attraction of a magnet field

A fresh pigskin sprayed with glycerol for moisturizing and lubrication is used as the substrate. A robotic arm holding a cylindric permanent magnet (N52, 15 mm in diameter × 20 mm in height) drives a 4-HCC on the pigskin substrate. The motion performance for HCC is tested by varying the distance between the magnet and the capsule (1.0 mm, 1.5 mm, 1.6 mm, 1.7 mm, 1.8 mm, and 1.9 mm) and changing the movement speed of robotic arm (output powers at 10%, 20%, 30%, 40%, 50%, 60%, 70%, 80%, 90%, and 100%). The relationship between end-effector velocity and power output of robotic arm is shown in Fig. S29.

Induction heating performance in a high-frequency alternating magnetic field

The induction heating of MLMs with different magnetic particle mass fractions (0wt%, 10wt%, 20 wt%, and 30 wt%) are tested under different distances from an electromagnetic coil. During the

experiment, a water-cooled hollow copper tube is connected to the magnetic induction device (50 kHz, 10 kW) to activate a high-frequency alternating magnetic field. The coil size and shape are shown in the Fig. S32. Bulky MLMs are not encapsulated by hydrogel coatings to capture accurate temperature changes with a temperature sensor. Furthermore, Ga-20% Fe@SiO_2 emulsion is prepared and placed above the electromagnetic coil for testing induction heating under the same experimental conditions, demonstrating the outstanding advantages of bulky MLM in magnetic induction heating.

In vitro cell toxicity test for HCC

We immerse HCCs in a 37°C standard DMEM medium to obtain leach liquors, where the extraction ratio is controlled to 0.2 g/mL. The leach liquors from HCC are dissolved in the DMEM to form different concentrations (25%, 50%, 75%, and 100% v/v). Normal human colon mucosal epithelial cells (NCM460), human gastric epithelial cells (GES-1), and human embryonic kidney cells (293T) with a density of 3000 cells per well are adherently cultured in the mixed DMEM/HCC with different concentrations. After co-culture for 24 h, CCK8 is used to measure cell viabilities. All of the tests are repeated six times to reduce errors.

Live/Dead cell staining assay

NCM460, GES-1 and 293T cell lines are seeded onto a 96-well plate at a density of 3000 cells per well, and cultured with the control group (DMEM) and 100% leach liquors for one day and three days. The cells are washed three times with PBS and stain with Live/Dead viability/cytotoxicity kit after the mediums are removed. Finally, the cells are washed with PBS several times, and staining results can be observed by a confocal microscope. Red areas and green areas represent dead cells and living cells, respectively.

Characterization of scald gastric tissue

Masson’s trichrome, hematoxylin and eosin staining (H&E) for gastric mucosal injury

Rabbits are euthanized after targeted thermal ablation operation. Stomach samples are promptly collected, opened along the greater curvature and rinsed with saline to remove the gastric contents in order to assess the extent of gastric damage. Thereafter, each stomach samples are fixed overnight with 4% formaldehyde, at 4℃. Samples are then processed for paraffin embedding, sectioning and staining according to standard histological (Masson’s trichrome, H&E), and observe by a morphologist who is blinded to the experiments.

Terminal-deoxynucleotidyl transferase mediated nick end labeling（TUNEL) assay

Stomach specimens are fixed with 4% formaldehyde, embedded in paraffin, and cut into 4 um thickness tissue sections and permeabilized for 30 min using a 0.1% Triton X-100 solution. The degrees of apoptosis in gastric tissues are detected by TUNEL staining using a One Step TUNEL Apoptosis Assay Kit (Beyotime Biotechnology Co., China) according to the manufacturer’s instructions. Briefly, these samples are washed in PBS for 3 times, following by incubation with the TUNEL reaction mixture containing terminal deoxynucleotidyl transferase (TdT)-mediated Fluorescein 5-isothiocyanate (FITC)-labeled dUTP for 1 h at 37 °C in the dark. After washing again in PBS for 3 times, nuclei are stained with 49, 6-diamidino-2-pheny-lindole (DAPI) (Sigma-Aldrich, St. Louis, MO, USA) for 10 min. Then, the TUNEL-stained slides are observed using a confocal

laser scanning microscope (Carl Zeiss, Germany). All the experiments were performed in triplicate.

Experimental detail of *ex vivo* and *in vivo* demonstrations

Ex vivo demonstration in fresh porcine stomach

One HCC is placed inside a fresh porcine stomach, and simulated stomach fluids are sprayed onto the stomach surface to mimic a real-life physiological environment. An cylindrical permanent magnet (N52, 15 mm in diameter × 5 mm in height) is used to control the HCC and to guide it to a specified location. Subsequently, the permanent magnet is replaced with an electromagnetic coil. With the coil positioned ~2 cm away from the HCC, a high-frequency alternating magnetic field (50 kHz, 10 kW) is activated to achieve rapid heating for the HCC. After the heating process is finished, the magnet replaces the electromagnetic coil to control the HCC to exit from the heating area.

In vivo demonstration in adult New Zealand rabbit model

This study aims to collect basic evidences for targeted thermal ablation operation using the proposed HCCs in live animals. Male New Zealand rabbits, weighing ~2 kg, are acquired from Kelian Rabbit Industry Professional Cooperative. The diameter of applied HCC is optimized to around 6 mm for easy oral administration. Initially, MLMs encapsulated in hydrogel coatings are in solid form during preservation. Thus, the HCC needs to be heated to ~38 ℃ to match the body temperature of rabbit and to melt the inner MLMs. The transition from solid to liquid significantly reduces the stiffness of capsule from GPa to kPa, preventing mechanical damage when the capsule interacts with the gastrointestinal tract. The unanesthetized rabbit then ingests the soft HCC orally with the assistance from experimenters. After about 4 h, the rabbit is anesthetized to enable stable imaging, including X-ray and CT scans, to confirm the position of HCC. Based on this positional information, a robotic arm equipped with a spherical permanent magnet (N48, 25 mm in diameter) can attract the inner HCC and guide it along a predetermined path. Upon reaching the target location, the permanent magnet is replaced by an electromagnetic coil connected to an induction heating device. After further confirming the position of HCC using X-ray imaging, the coil is aligned with the center of capsule to ensure proximity. A high-frequency alternating magnetic field (50 kHz, 10 kW) is then activated to heat the inner HCC wirelessly. This heating process, lasting about 10 seconds, is followed by a 10-second cooldown period before restarting the device. The cycle is repeated five times to ensure successful thermal ablation in the rabbit stomach model. Subsequently, the ingested HCC is retrieved through dissection to examine the structural integrity of the hydrogel coating, and check for any leakage of liquid metal inside the body. Additionally, the heat-treated gastric tissues are evaluated through observation and histological stain analysis to assess the effectiveness of wireless heating.

Supplementary Table

Table S1. Comparison between various flexible matrix[3]**.**

Flexible matrix	Density (g/cm^3)	Melting point (°C)	Electrical conductivity (S/m)
Water	0.997	0	0.05501×10^{-6}
Silicone oil	0.963	-60 - 0	10^{-12} - 10^{-14}
Hydrogel （e.g. polyacrylamide hydrogel）	~1	/	(10 - 80) × 10^{-6}
Silicone rubber	0.95 - 1.4	/	10^{-12} - 10^{-14}
Polyvinyl alcohol-borax slime	1 - 1.10	/	~0.3
**Gallium**	**6.077**	**29.8**	**3.8×10^{6}**

Table S2. Material classification for magnetic thermal ablation[4–7]**.**

Material classification for magnetic ablation	Modulus compared with body tissue (10^2 to 10^7 Pa)	Heat efficiency	After-use residue	Targeting ability
Millimeter-scale solid metal (e.g. Nickle-Copper alloy)	$>10^7$ Pa	Middle, eddy-current heating	No	Poor
Magnetic micro/nano-particles (e.g. Iron oxide particle and Mn-Zn ferrite nanoparticle)	$>10^7$ Pa, but the size of micro/nanoparticles is enough small to avoid mechanical mismatch	Relatively low, magnetothermal heating	Yes	Good
Liquid metal (e.g. Gallium and gallium-based alloy)	$<10^2$ Pa	Middle, eddy-current heating	Yes	Poor
Magnetic liquid metal (e.g. Gallium-iron particle compound and Gallium-Fe_3O_4 particle compound)	$<10^2$ Pa	High, both of eddy-current heating and magnetothermal heating	Yes	Good
**Hydrogel-coated containment capsule of magnetic liquid metal (HCC in our work)**	**10^4 to 10^5 Pa**	**High, both of eddy-current heating and magnetothermal heating**	**No**	**Good**

Table S3. Comparison and schematic illustrations between our technique and existing encapsulation methods for macroscopic liquid metal droplets[8–12]**.**

Encapsulation technologies	Time for encapsulation	Condition for encapsulation	Interfacial adhesion	Coating uniformity	Coating continuity	Functionality	Schematic illustration
Dip coating using silicone precursor solution	A few hours	~60°C	√	×	√	×	
Mold process method using silicone precursor solution	A few hours	~60°C	√	√	√	×	
Dip coating using hydrogel precursor solution	A few hours	Ultraviolet or high temperature	√	×	√	√	
Mold process method using hydrogel precursor solution	A few hours	Ultraviolet or high temperature	√	√	√	√	
Wrapping using thin film	A few minutes	Mechanical sealing, high temperature, or additional adhesive	×	×	√	×	
Rolling coating using powers without self-gelation ability	<1 min	/	√	√	×	√	
**Rolling coating using our lyophilized hydrogel powers**	**<1 min**	**Few water to hydration**	**√**	**√**	**√**	**√**	

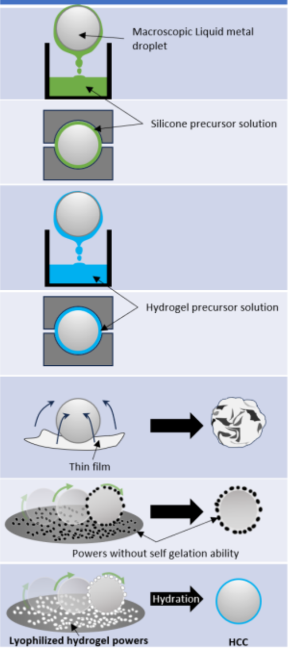

Supplementary Figure

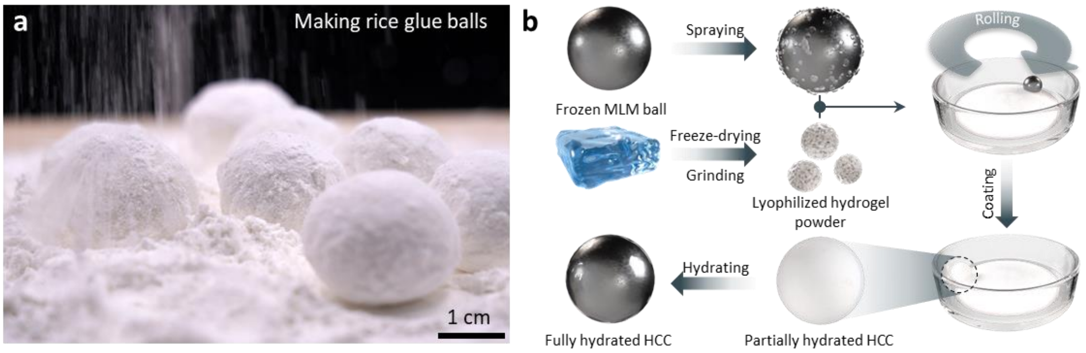

Fig. S1. Concept and preparation of hydrogel-coated containment capsule (HCC) of magnetic liquid metal. a, Encapsulating fillings with flour to form rice glue balls (also called Tangyuan, a Chinese traditional food). **b**, Schematic illustration on the fabrication process of HCCs.

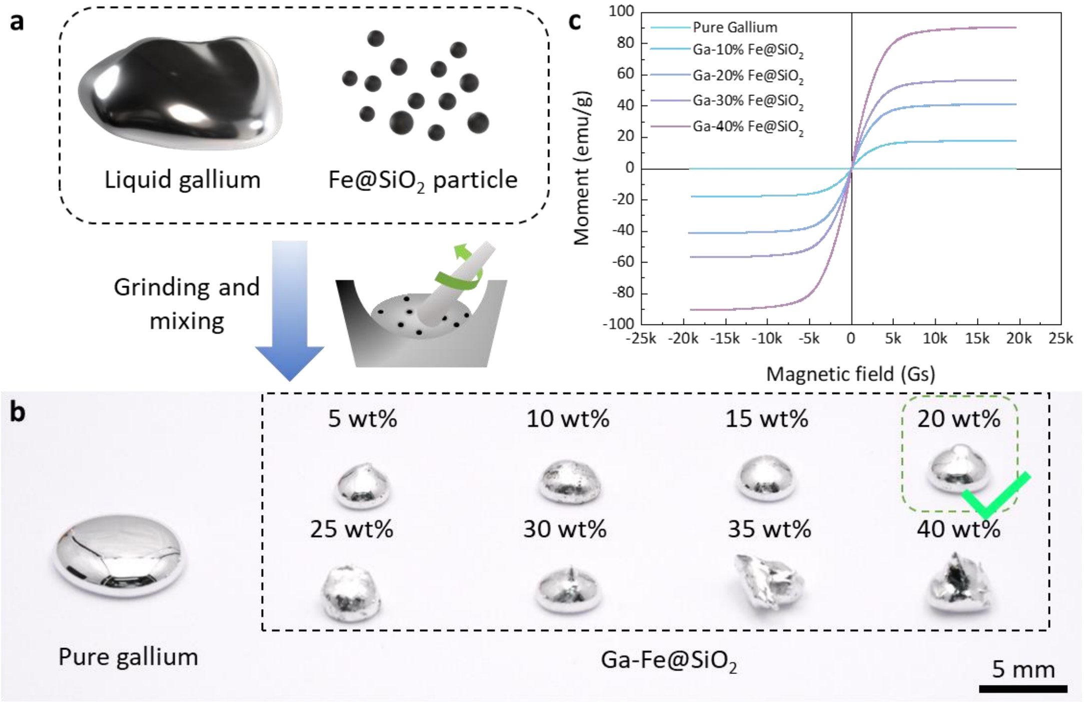

Fig. S2. Fabrication and magnetic property of magnetic liquid metals (MLMs). a, Schematic illustration of fabrication process for Ga-Fe@SiO_2. **b**, Optical image of MLMs with different Fe@SiO_2 mass ratios, where Ga-20wt% Fe@SiO_2 composites are selected to be used considering magnetism and fluidity. **c**, Magnetic performance of MLMs with different Fe@SiO_2 mass ratios.

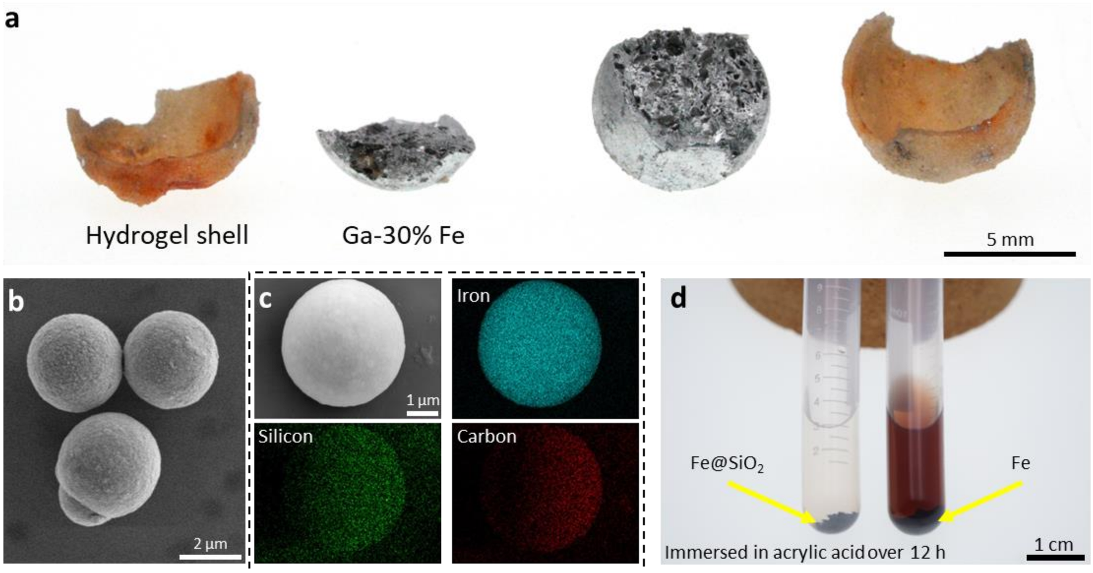

Fig. S3. Surface modification to avoid the corrosion between iron particles and acrylic acid. a, Disassembly of HCCs, highlighting the transformation of the transparent coating to yellow due to the reaction between acrylic acid and embedded iron particles. **b**, Scanning electron microscope of Fe@SiO_2. **c**, Energy disperse spectroscopy images of Fe@SiO_2. **d**, Comparison between bare Fe microparticles and Fe@SiO_2 microparticles immersed in acrylic acid solutions over 12 h, showing the protective efficacy of SiO_2 shells.

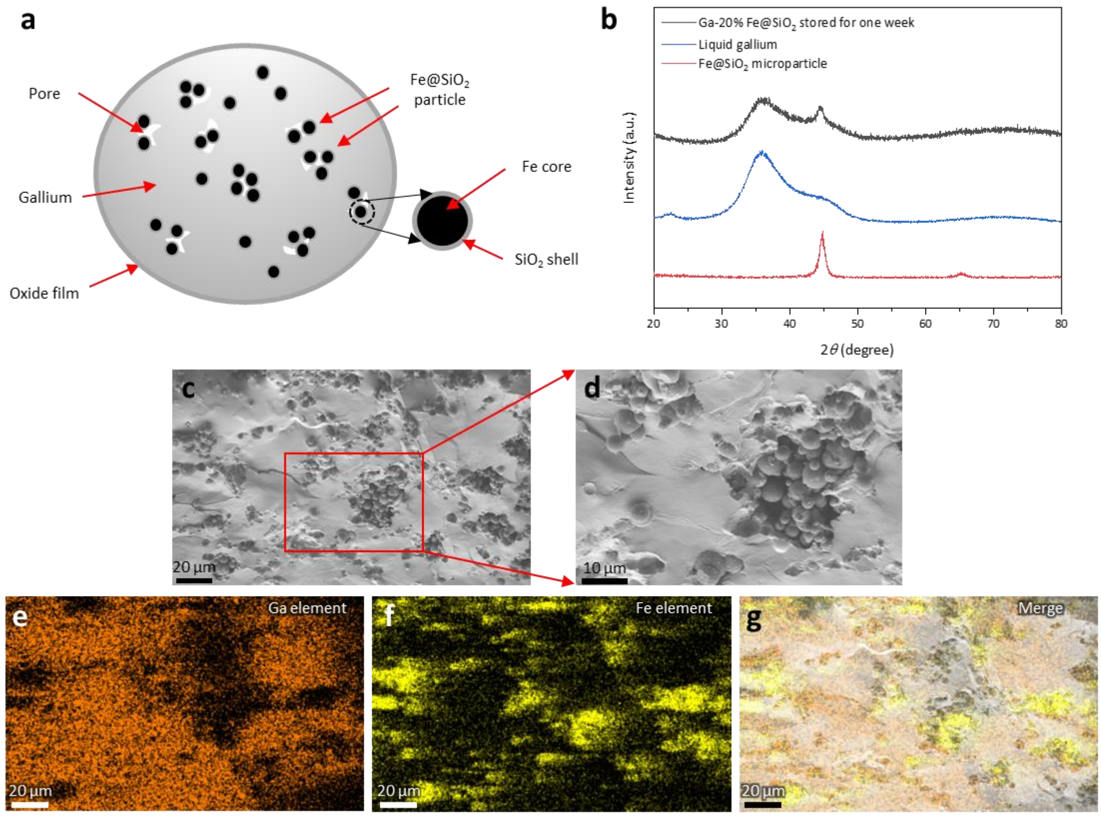

Fig. S4. a, Schematic diagram showing the inner structure of Ga-Fe@SiO_2 composites. b, X-ray diffraction patterns of Ga-20% Fe@SiO_2, liquid gallium, and Fe@SiO_2 microparticle stored for one week at room temperature. **c**, Scanning electron microscopy showing the cross-sectional microstructure of solidified Ga-20% Fe@SiO_2. **d**, Zoom-in image of red box in **c** highlighting the distribution of Fe@SiO_2 in gallium. Energy disperse spectroscopy images display the distribution of gallium (**e**) and iron elements (**f**), respectively, as referenced in **c**. **g**, Element mapping overlay showing the distribution of Ga and Fe elements.

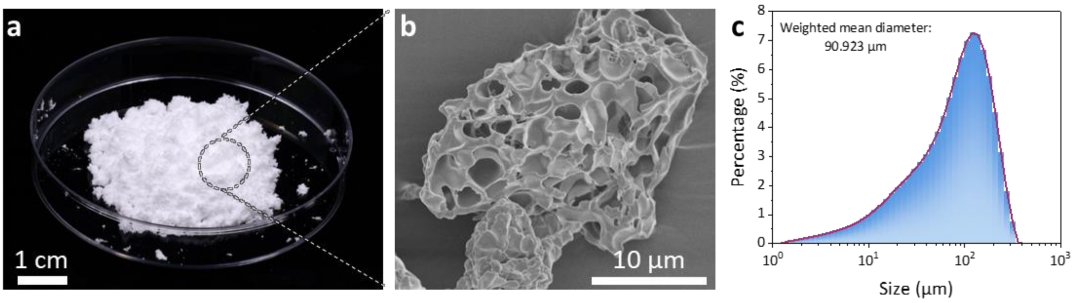

Fig. S5. Characterization for lyophilized hydrogel powders (LHPs). a, Optical image of LHPs. **b**, Scanning electron microscope image showing the morphology of LHPs. **d**, Size distribution of LHPs.

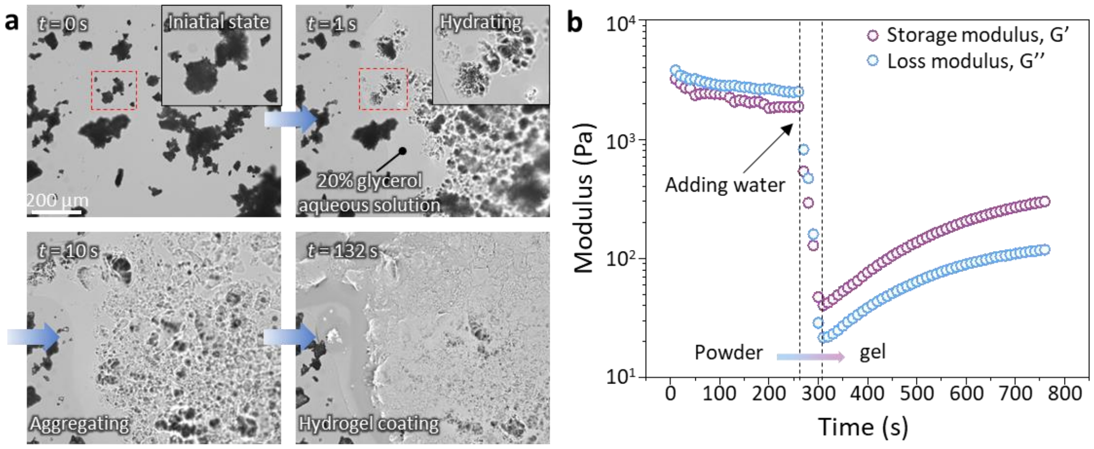

Fig. S6. Self-gelling process of LHPs. a, Optical microscope series capturing the self-gelling transformation of LHPs in glycerol aqueous solutions. **b**, Rheological evaluation of LHPs during hydration. Gelation occurs within 30 seconds when the storage modulus (G') curve intersects with the loss modulus (G'') curve.

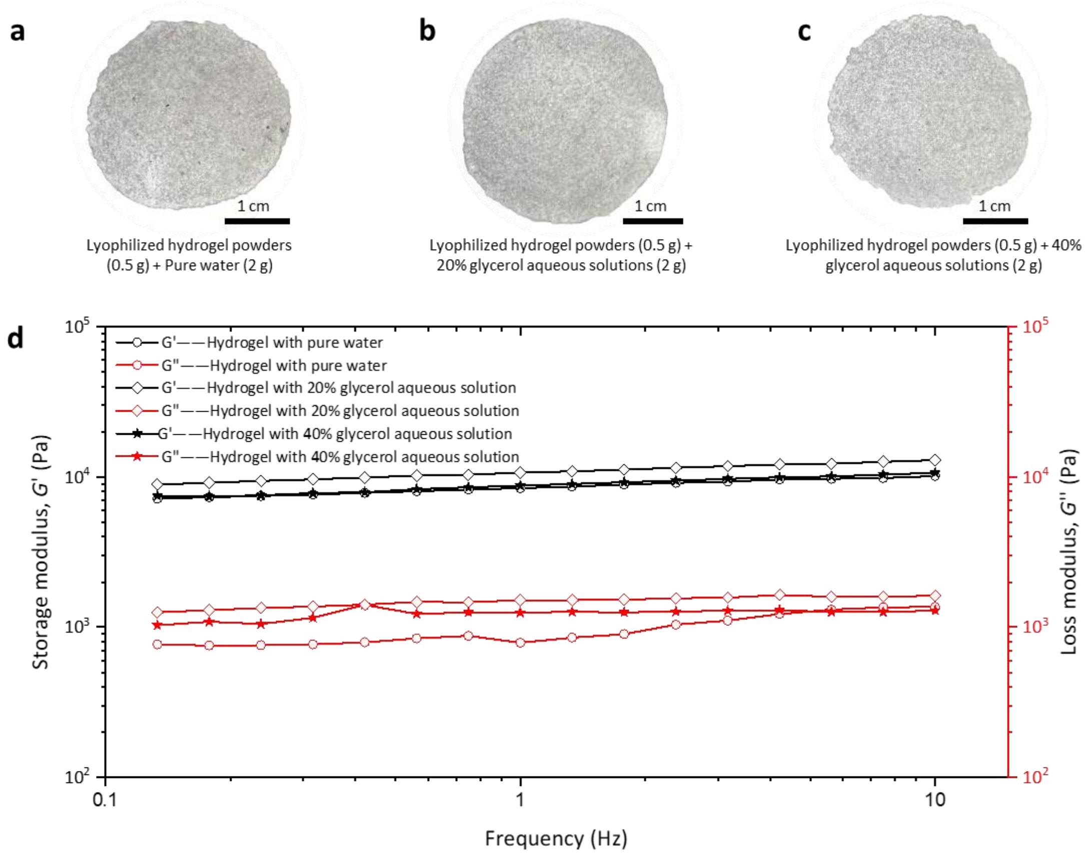

Fig. S7. **a**, **b**, and **c** show the morphologies of hydrogel samples hydrated with glycerol aqueous solutions containing 0%, 20%, and 40% glycerol, respectively. The solid content of above samples is controlled to 20%. **d**, Rheological performances of various hydrogel samples hydrated with glycerol aqueous solutions containing 0%, 20%, and 40% glycerol concentrations under various frequencies.

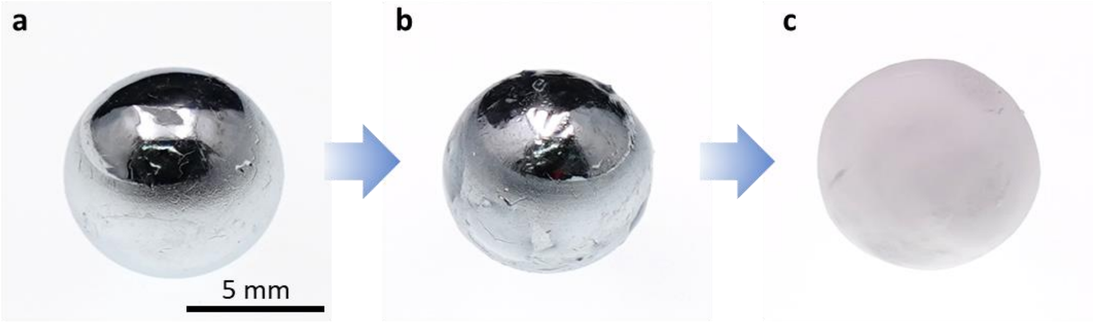

Fig. S8. a, Optical images showing appearances of solid MLM balls. **b**, Solid MLM balls sprayed with glycerol aqueous solutions. **c**, Partially hydrated HCCs.

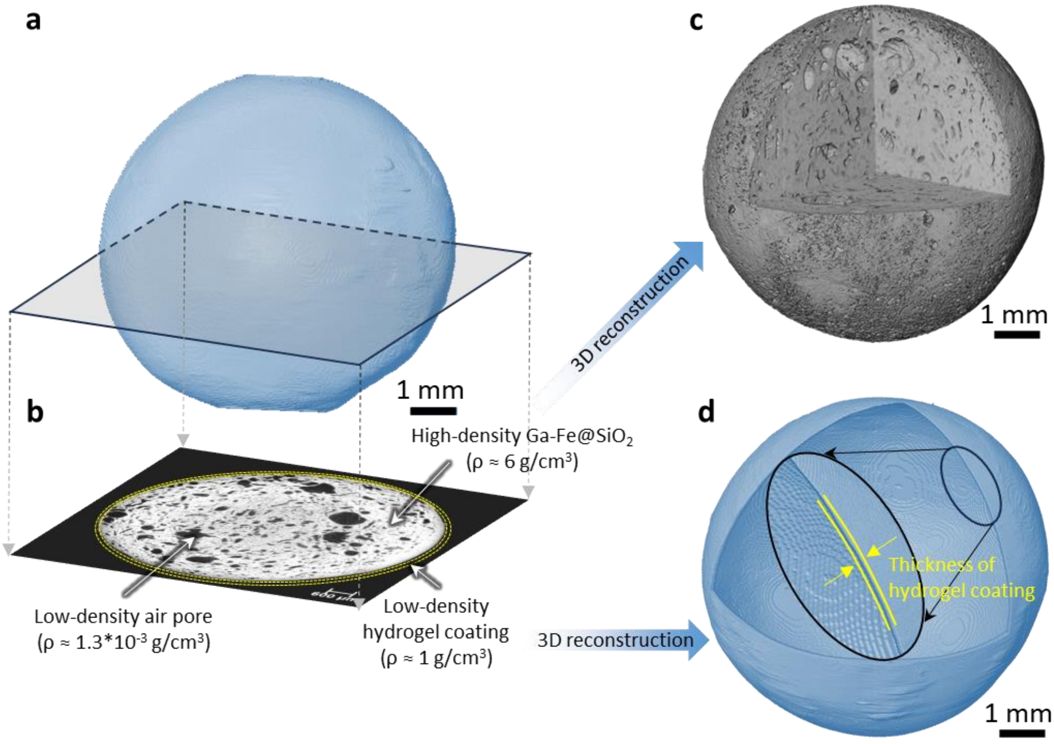

Fig. S9. Three-dimensional (3D) reconstruction from micro computed tomography (micro-CT) images showing the entire morphology of 1-HCC (**a**), sectional X-ray image showing the inner structure including low-density air pore, low-density hydrogel coating, and high-density Ga-Fe@SiO_2 (**b**), 3D reconstructions showing the inner structures of Ga-Fe@SiO_2 core (**c**), and monolayer hydrogel coating (**d**).

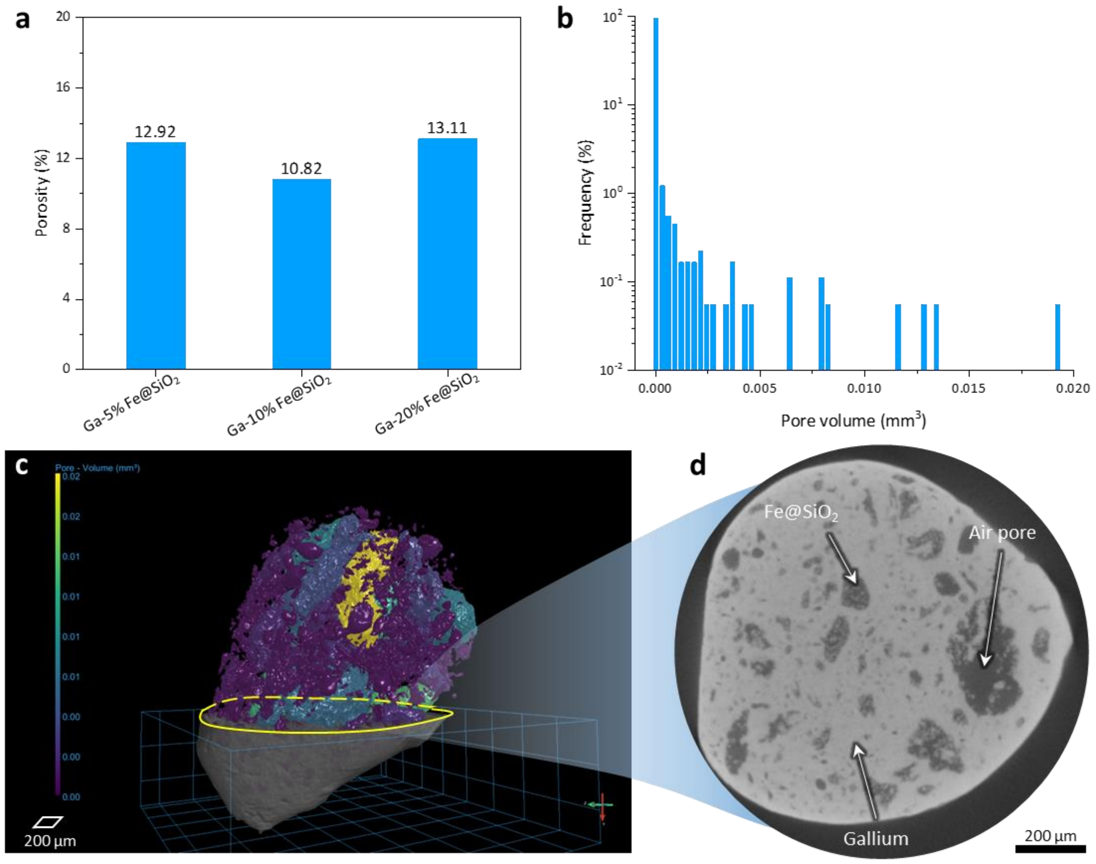

Fig. S10. a, Inner porosity as function of Fe@SiO_2 content in Ga-Fe@SiO_2 composites. **b**, 3D reconstruction for Ga-Fe@SiO_2 composites to characterize the distribution and size of inner pores. **c**, Sectional image showing the distribution of gallium, air pore, and Fe@SiO_2. **d**, The volumetric distribution ratio of internal pores.

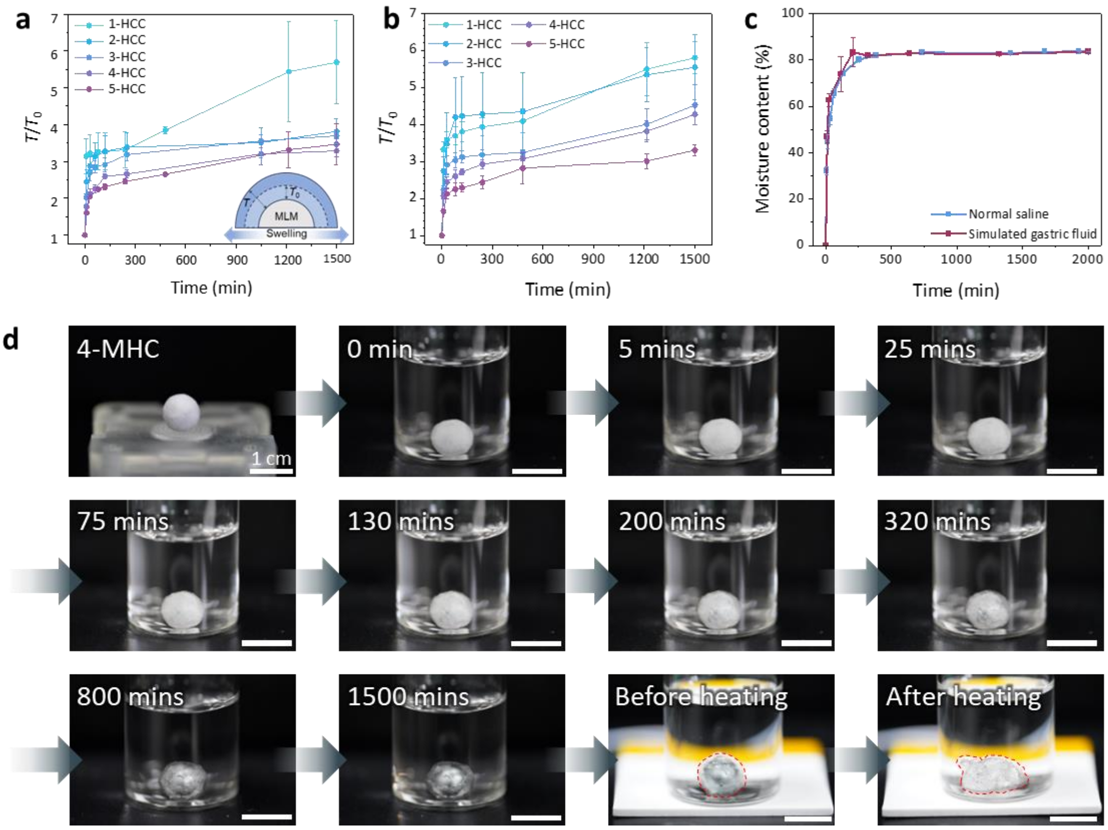

Fig. S11. Swelling behavior of hydrogel coating and the morphology change of HCCs after melting. a, Thickness evaluation of hydrogel coating on MHC with different layers in normal saline (NS) solutions, where T is the current thickness and T_0 is the initial thickness of hydrogel coating. **b**, Thickness evaluation of hydrogel coating on HCC with different layers in simulated gastric fluid (SGF). The 1-HCC reaches to the swelling equilibrium state faster than other HCCs with more hydrogel coating layers due to rapid diffusion for water. The violent change for thickness of 1-HCC is attributed to wrinkles on hydrogel surface. **c**, Moisture content changes over time for dried p(AAc-*co*-TMSPMA) hydrogel immersed in NS and SGF. **d**, Swelling process of a 4-HCC immersed in NS at room temperature and the morphological change for swelled HCC after heating. Data are presented as mean ± standard deviation (s.d.); n = 3 independent experiments.

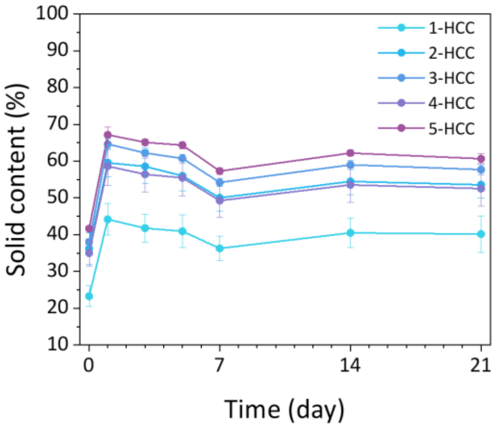

Fig. S12. Moisture retention capability of HCCs with different hydrogel coating layers for 3 weeks (in ~25 ℃, ~60% RH). The average solid content is ~60% when the system is in equilibrium. Data are presented by mean ±s.d.; n = 3 independent experiments.

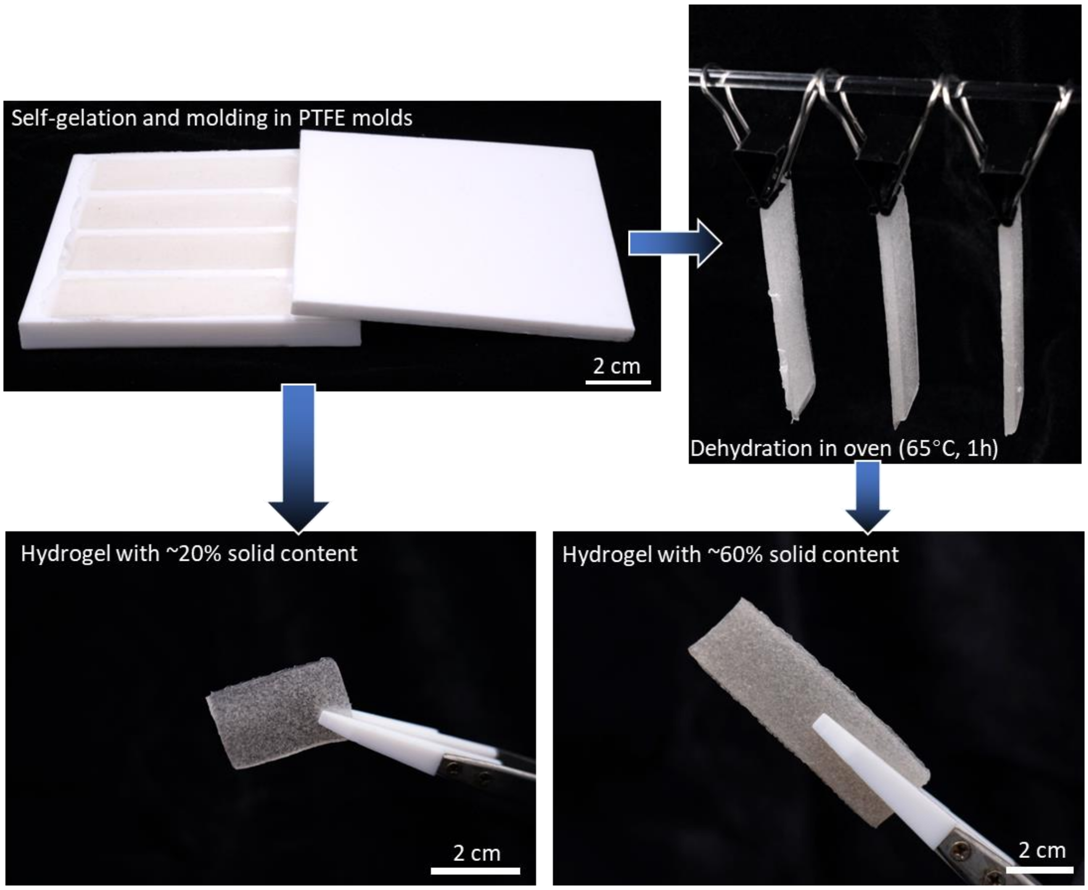

Fig. S13. Fabrication process for tensile specimens of hydrated hydrogels with varied solid contents. The solid content within the hydrogels is modulated through controlled dehydration times in an oven.

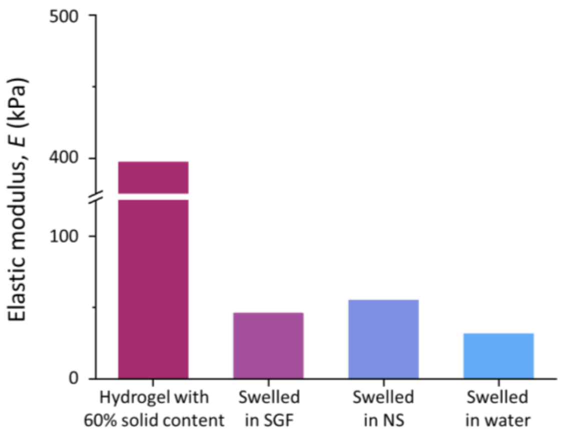

Fig. S14. Elastic moduli of ~60% solid content hydrogel samples, and hydrogel samples swelled in SGF, NS and pure water for 24 h.

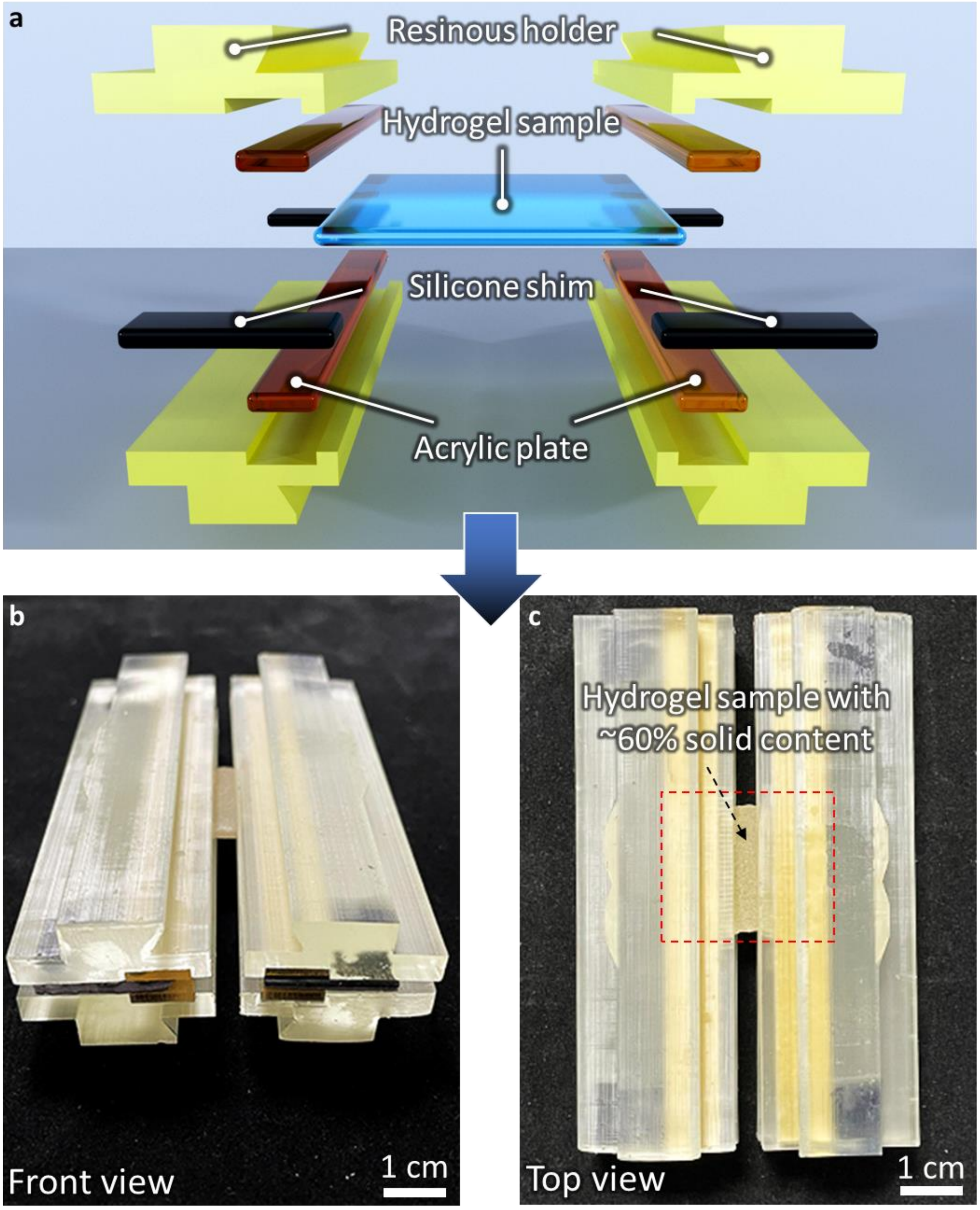

Fig. S15. Overview of the custom-designed tensile fixture. a, Schematic illustration of the tensile fixture for testing hydrogel toughness. This fixture incorporates resinous holders crafted via photopolymerization 3D printing, designed to interface seamlessly with static tensile machine grips. Silicone shims are employed to mitigate over-extrusion in soft hydrogel samples. Acrylic plates ensure sample stability, prevent slippage, and allow for straightforward sample replacement. The front view (**b**) and top view (**c**) of the custom-designed tensile fixture.

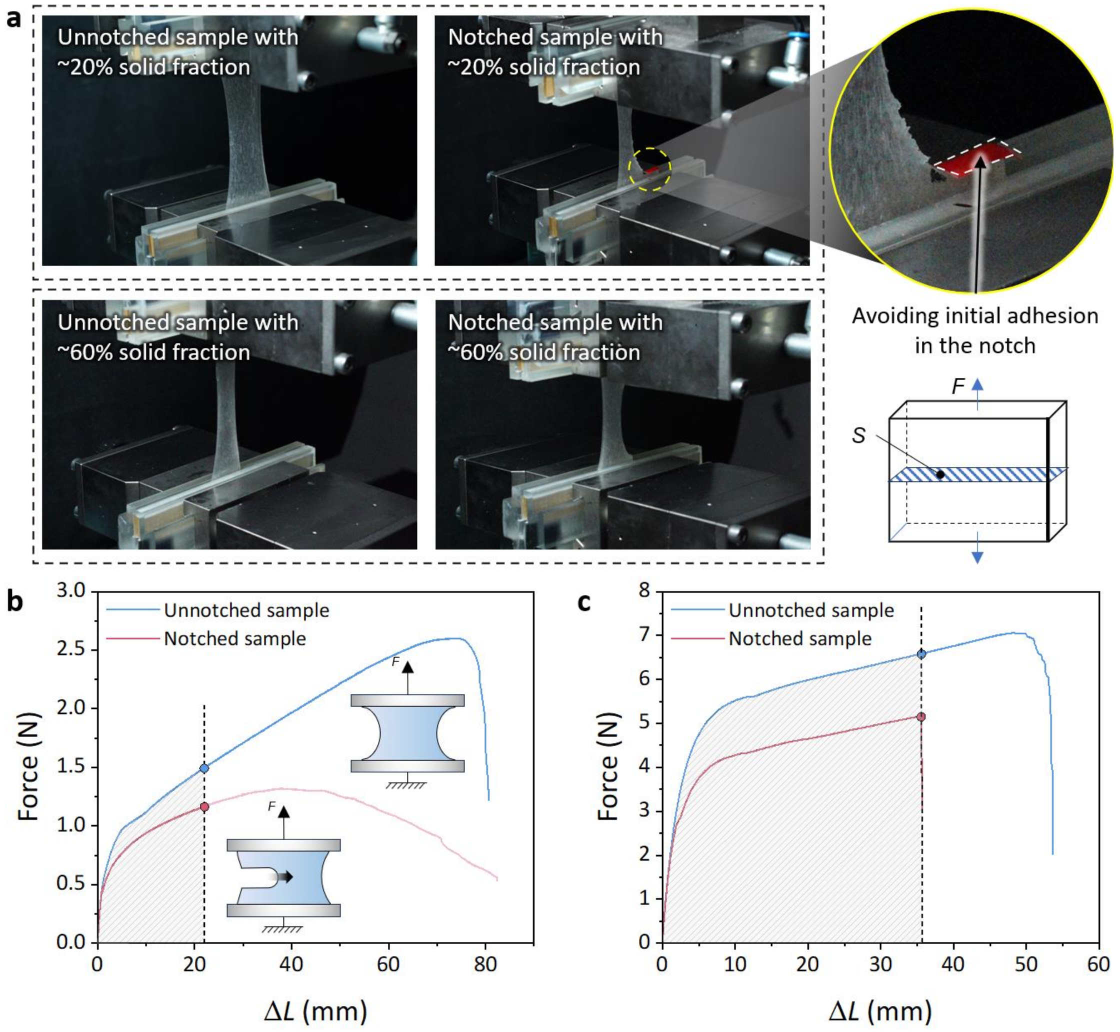

Fig. S16. Demonstration of toughness measurement for hydrated hydrogel samples. a, Photographs showing test processes for toughness of samples with ~20% solid fraction and with 60% solid fraction. The added red plastic sheet can avoid undesired initial adhesion in the notch in the zoom-in insert image. The cross-sectional area of original tensile sample is defined by *S*. **b**, Force-length curves of unnotched and notched samples with ~20% solid fraction. **c**, Force-length curves of unnotched and notched samples with ~60% solid fraction. The area beneath the force-length curve represents the work done by the force on the unnotched sample, U(*L*). The fracture energy (toughness) is calculated from U(*L*)/*S* [13].

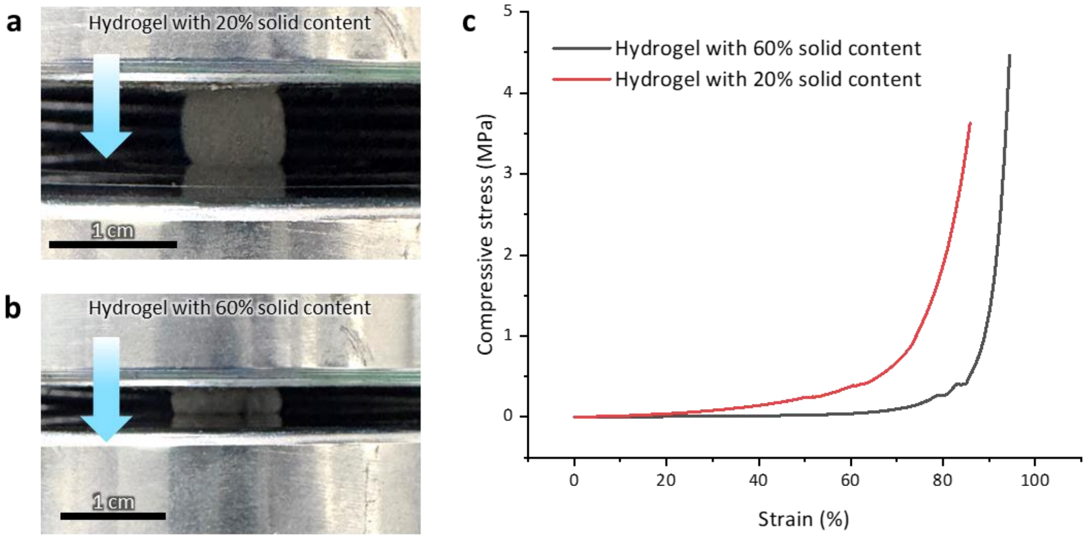

Fig. S17. **a**, Compression process of hydrogel with 20% solid content. **b**, Compression process of hydrogel with 60% solid content. **c**, Compressive stress-strain curves of hydrogel with 20% solid content and 60% solid content.

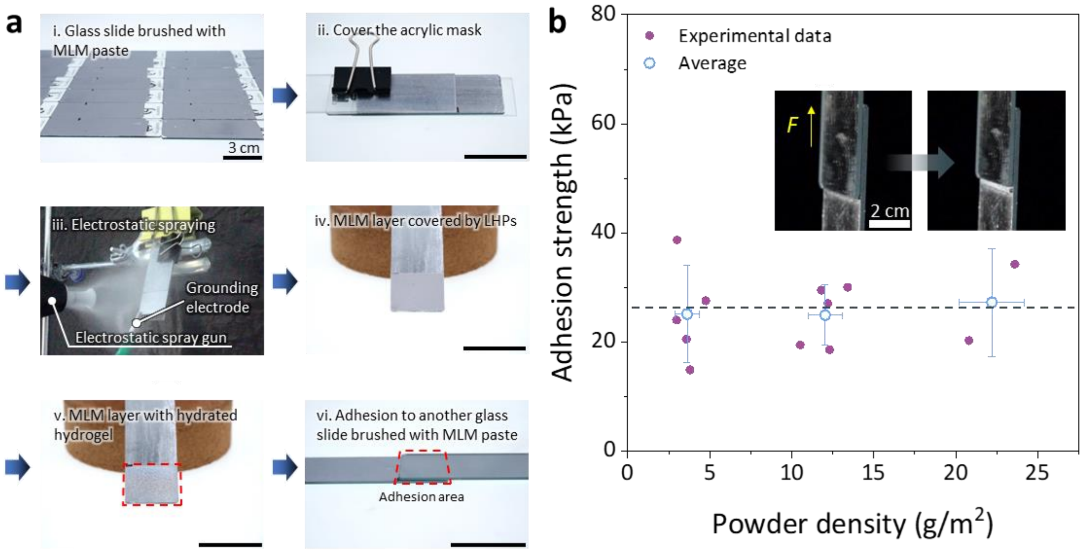

Fig. S18. Demonstration of measurement for adhesion strength of hydrated LHPs. a, A process to bond two pieces of glass brushed with MLM layers together using hydrated LHPs, where an electrostatic spraying method is used for controllability of powder density. **b**, Adhesion strength as a function of LHP density, where the horizontal dashed line represents the average of adhesion strength. The inset images depict the damage to the adhesive layer between two glass slides under external force.

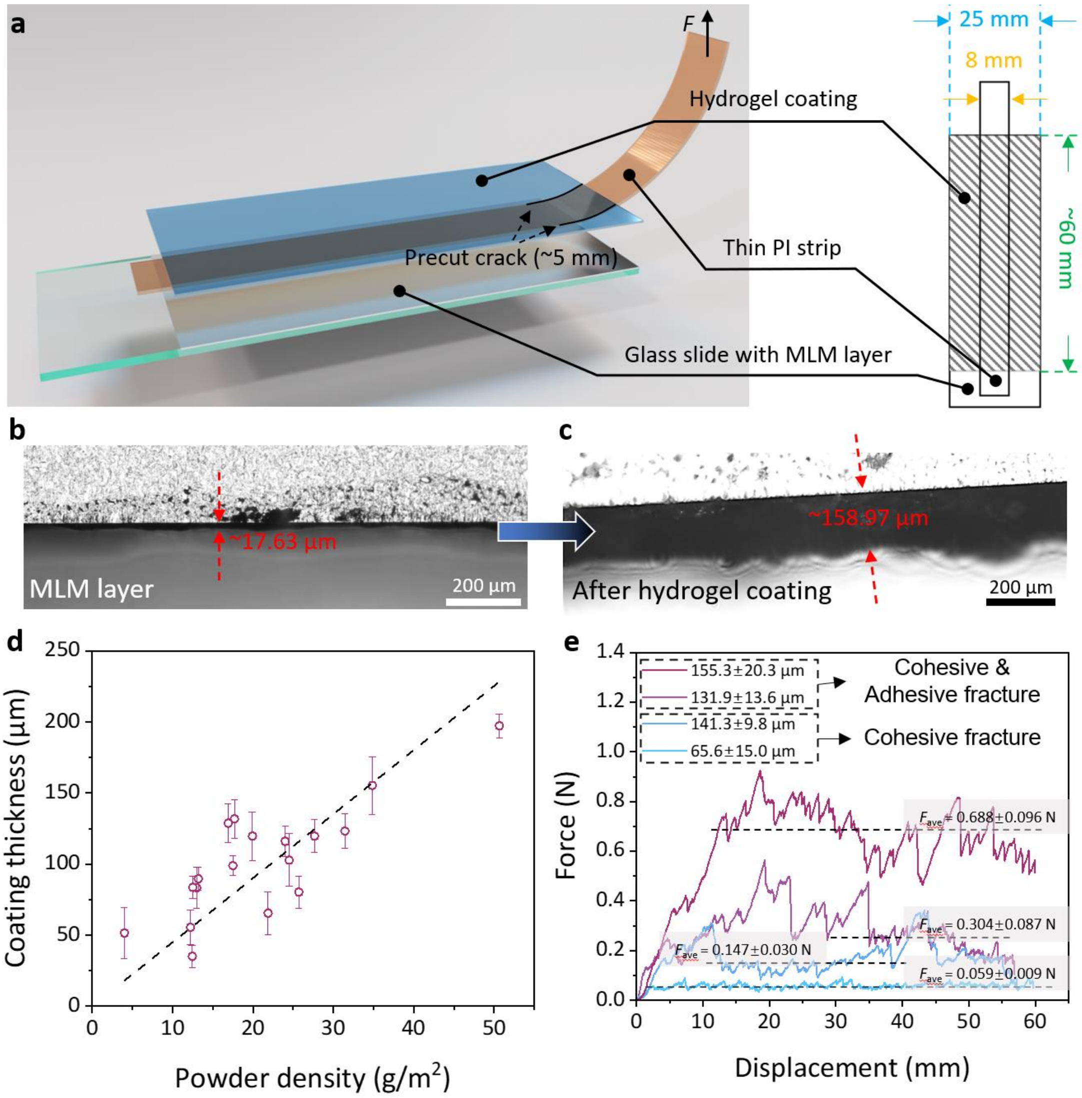

Fig. S19. Measurement for interfacial toughness of hydrogel coating on MLM layers. a, Schematic illustration of the 90°-tearing test for hydrogel coatings on the solidified MLM substrates. Optical microscopic images showing the thickness of MLM layer brushed on a glass slide (**b**) and the thickness of hydrogel coating on the MLM layer (**c**). **d**, Thickness change of hydrogel coating as a function of the LHP density. Data are mean ± s.d.; Thickness measurements are performed across 10 distinct regions within a single sample. **e**, Force-displacement curves of 90°-tearing test under different thicknesses of hydrogel coating, where the black dashed lines represent the average forces in plateau stages.

Here, we simply evaluate the data in **Fig. 2j.** The interfacial toughness, denoted by the energy release rate G, is assessed using a 90°-tearing test on hydrogel coatings on MLM layers. This method involves a pre-inserted 3-μm thick polyimide film under the hydrogel layer, which facilitates the bilateral tearing of the coating at a controlled speed as the film is lifted. The energy release rate G is calculated as $F/2h$, where F is the average force in the tearing stick-slip dynamics and h is the thickness of hydrogel coating[14]. Here, two fracture models exist, including the cohesive fracture model (data points in blue area and the experiment photo in the bottom right insert) and the cohesive & adhesive fracture model (data points in purple area and the experiment photo in the upper left insert). For thin coating, direct tearing at the front indicates cohesive fracture. New

interfaces are generated only in the thickness direction of the hydrogel coating upon tearing along both sides of polyimide strip in the cohesive fracture model. The calculated G from $F/2h$ reflects the interfacial bonding of hydrogel coating (425.3 J/m^2). For thicker coating, the hydrogel coating undergoes periodic large stretch and sudden rupture at the tear front, coupled with debonding between hydrogel and substrate near the tear front (the cohesive & adhesive fracture model). The front of tearing tends to appear an obvious triangle due to the stretch of hydrogel coating and deadhesion between hydrogel and MLM substrate. The coexistence of adhesive and cohesive fracture results in the formation of additional interfaces between the MLM layer and the coating. Therefore, the coupling of different fracture models (in the purple area) inflates the G from $F/2h$ that only considers the tearing energy. However, the tendency still implies the enhanced resistance to fracture and debonding as the powder dosage increases.

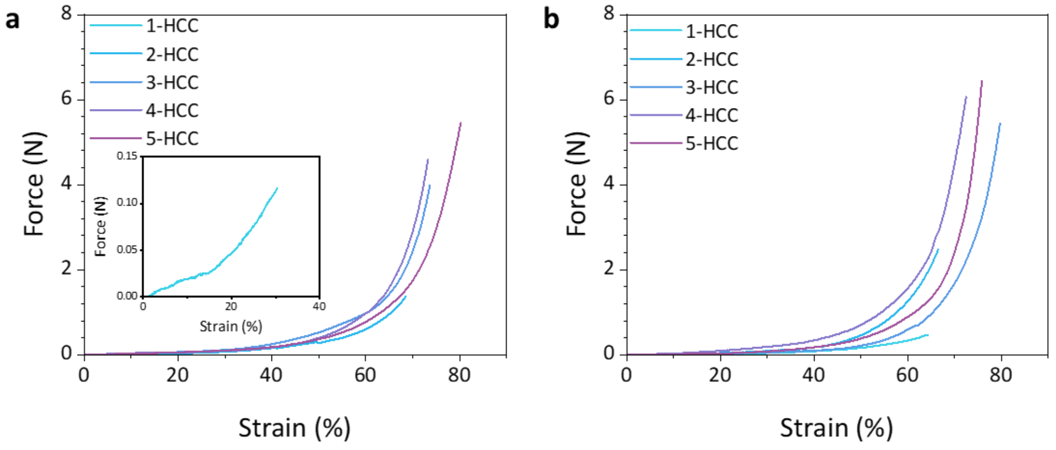

Fig. S20. Force-strain curves of HCCs with different hydrogel layers immersed in NS (**a**) and in SGF (**b**) for 24 h. The insert in **a** shows the force-strain curve of the 1-HCC immersed in NS for 24 h.

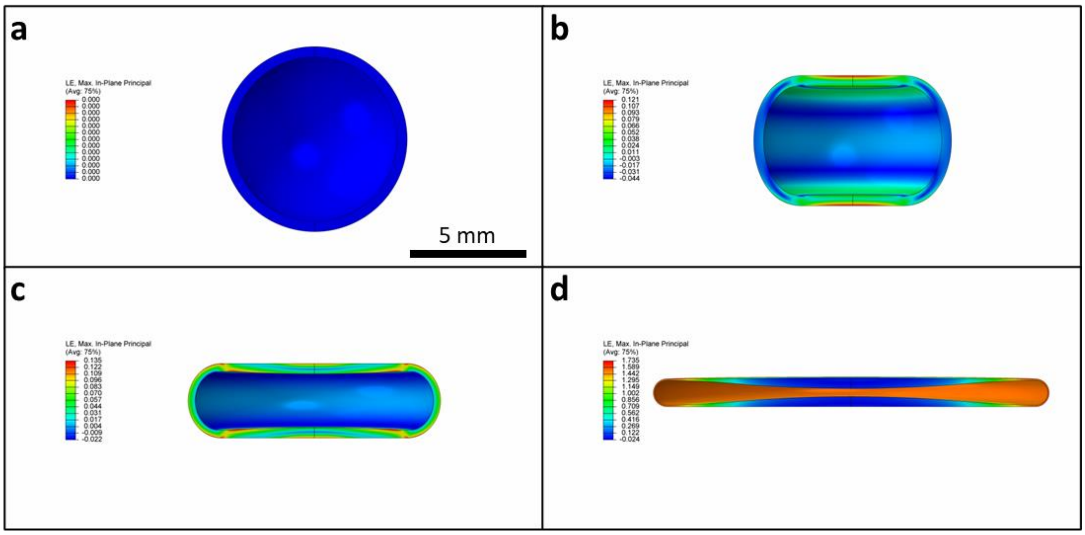

Fig. S21. Finite element analysis for a 4-HCC with 60% solid content hydrogel coatings under 0% compression strain (**a**), 30% compression strain (**b**), 60% compression strain (**c**), and 85% compression strain (**d**).

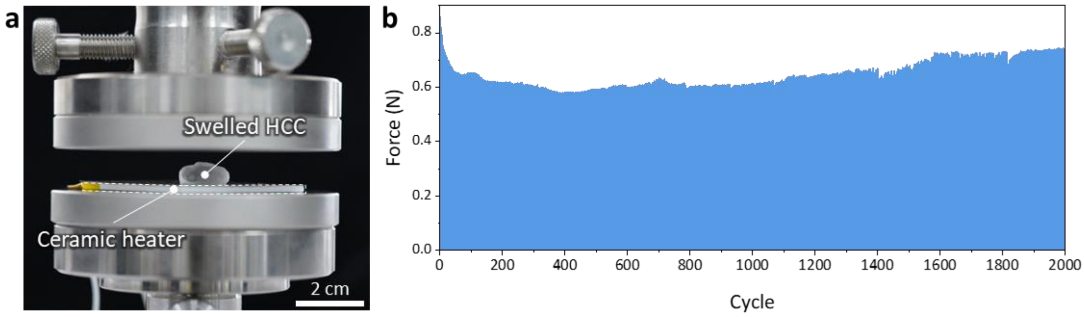

Fig. S22. Measurement for cyclic compressibility of a swelled 4-HCC. a, Optical image showing a compression test conducted on a 4-HCC swelled in SGF for 24 h, while its temperature at ~50 ℃ using a ceramic heater. **b**, Cyclic compressibility for the swelled 4-HCC, with compression deformation rate controlled at 40%. The increase of the force upon compression mainly attributes to the dehydration of swelled hydrogel coating.

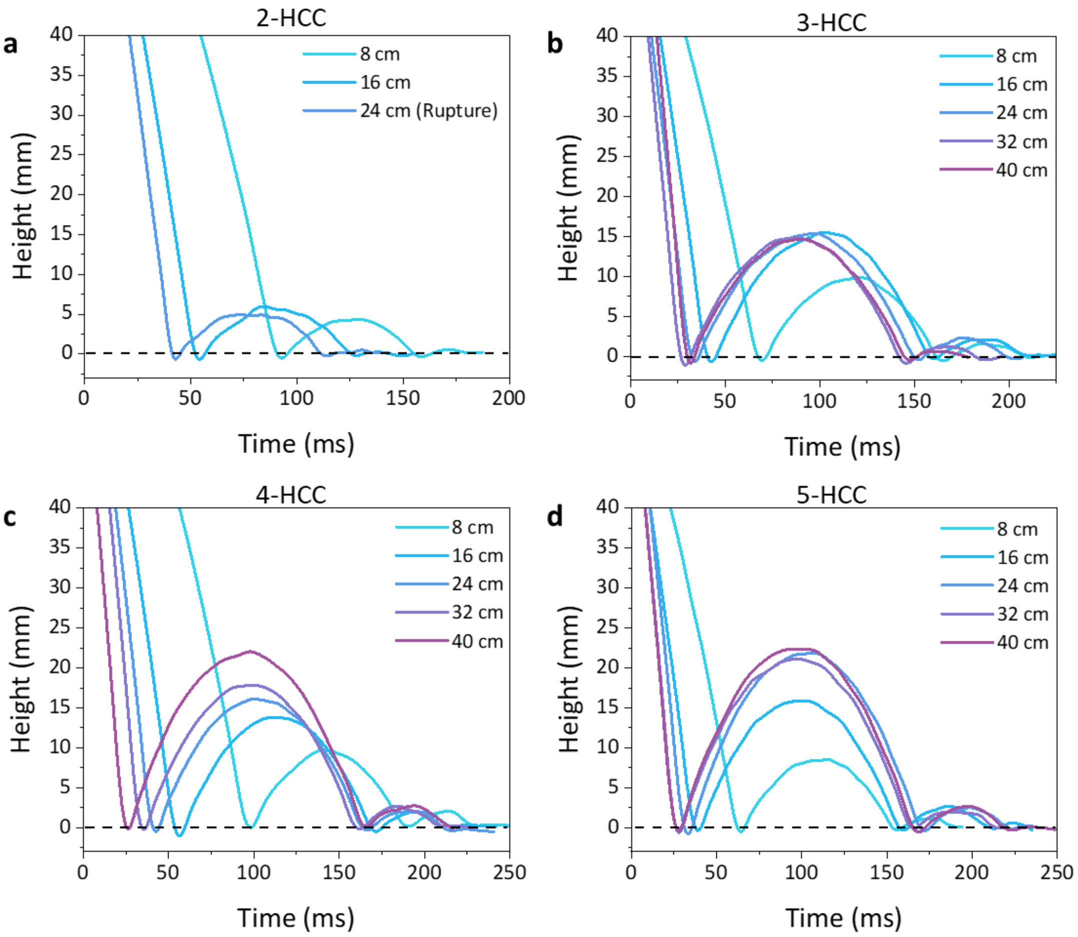

Fig. S23. Vertical height changes over time for 2-HCC (**a**), 3-HCC (**b**), 4-HCC (**c**), 5-HCC (**d**) falling from different heights. The black dashed lines represent the ground level. These HCCs are encapsulated by ~60% solid content hydrogel coatings.

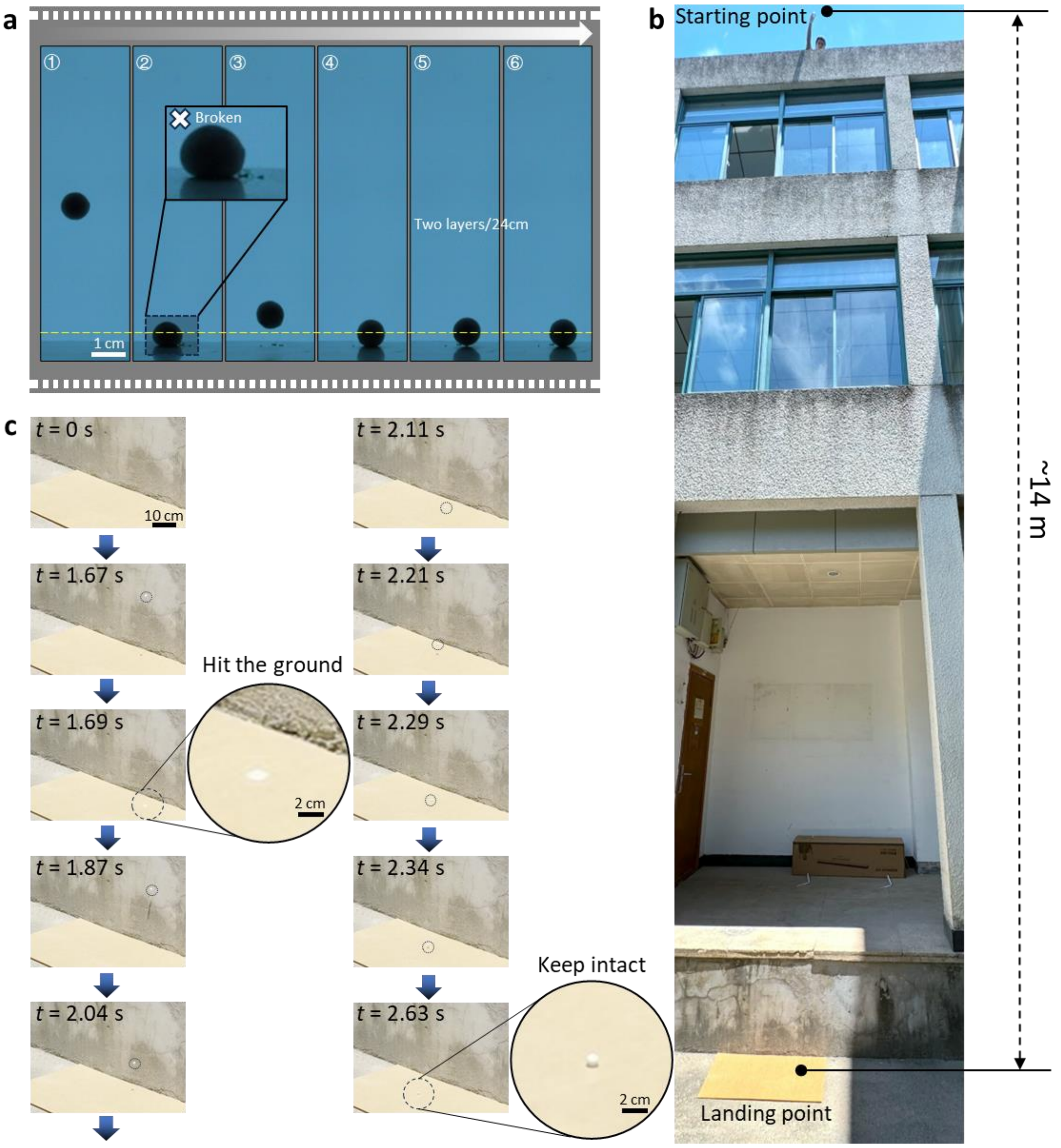

Fig. S24. Tests for impact resistance of HCC. a, Keyframes extracted from high-speed photography capturing a 2-HCC falling from a height of 24 cm. **b**, A 4-HCC fell from a height of 14 m. **c**, The falling process of the 4-HCC was captured at a height of 14 m, showing its strong impact resistance.

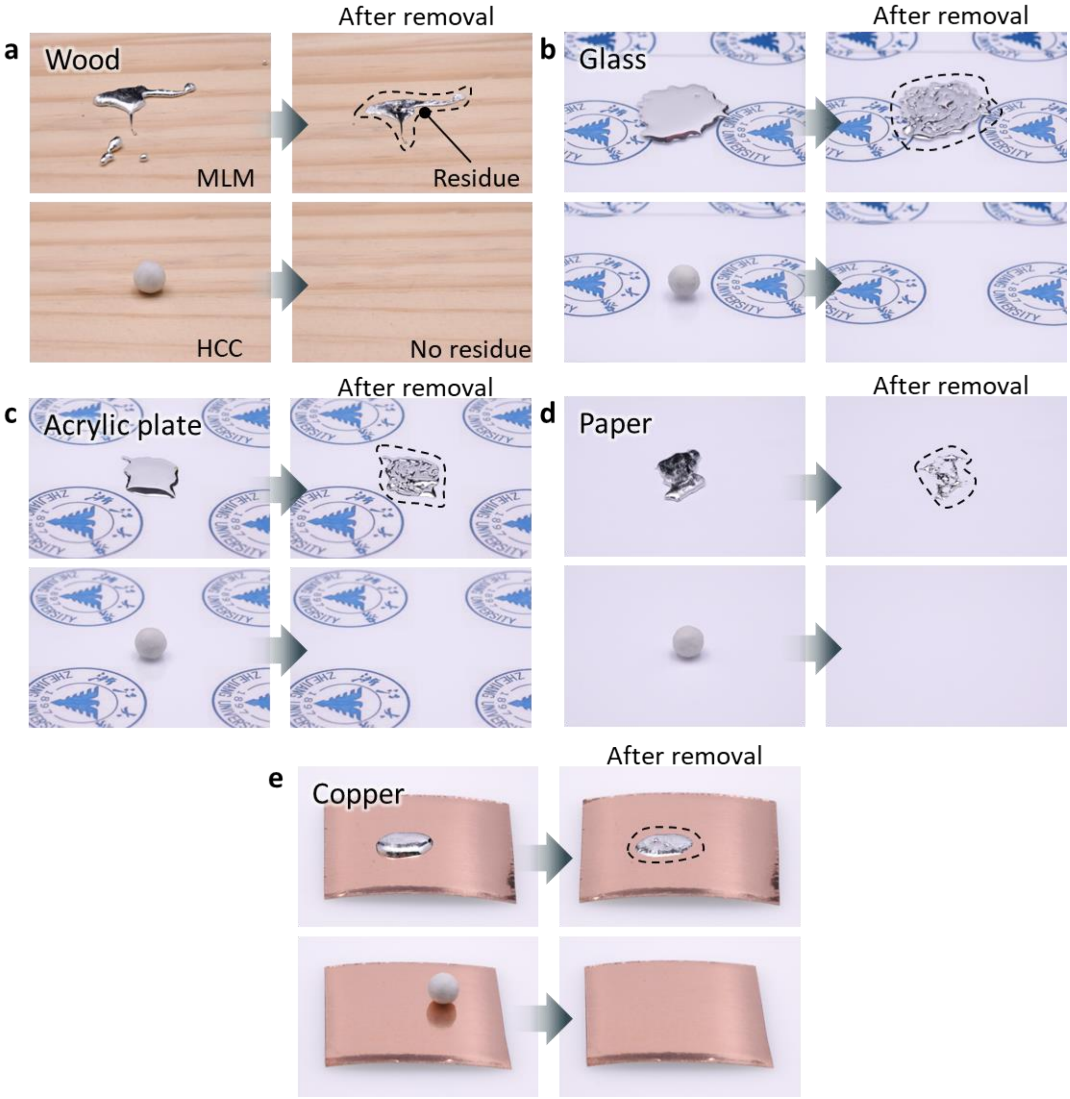

Fig. S25. Comparison of the residual properties between MLMs and HCCs on different substrates including the wood (**a**), glass (**b**), acrylic plate (**c**), paper (**d**), and copper (**e**).

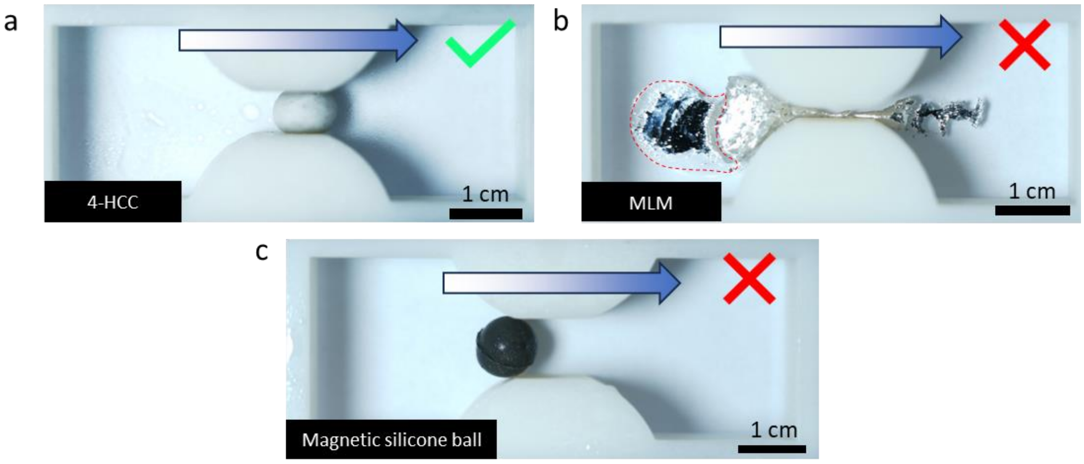

Fig. S26. The process for 4-HCC (**a**), MLM droplet (**b**), and magnetic silicone ball (**c**) to pass through narrow channels under the manipulation of external magnet, where the arrow indicates the motion direction.

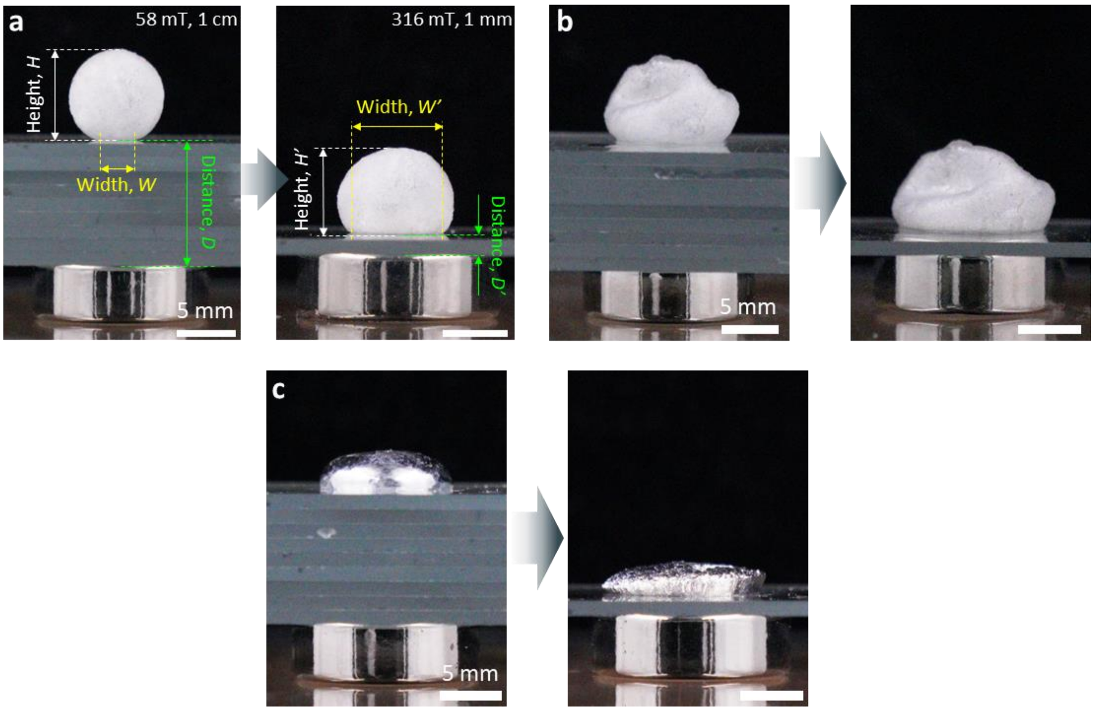

Fig. S27. Optical images showing active deformation performances of HCCs (**a**), swelled HCCs (**b**), and bare MLMs (**c**) in different magnetic flux densities.

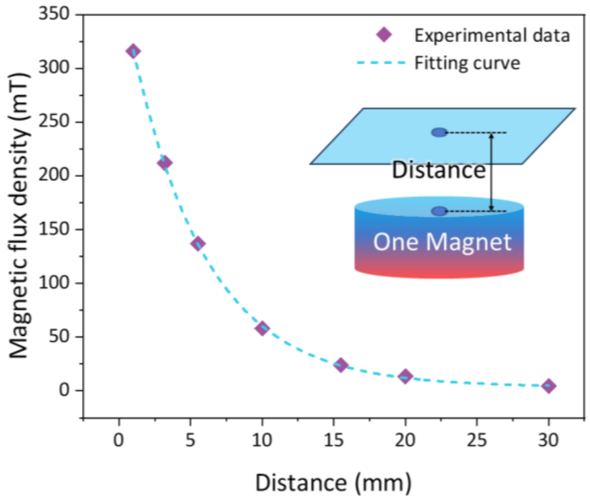

Fig. S28. Magnetic flux density versus the distance between a cylindrical permanent magnet (N52, 15 mm in diameter × 5 mm in height) and sample. The insert depicts the definition for the distance.

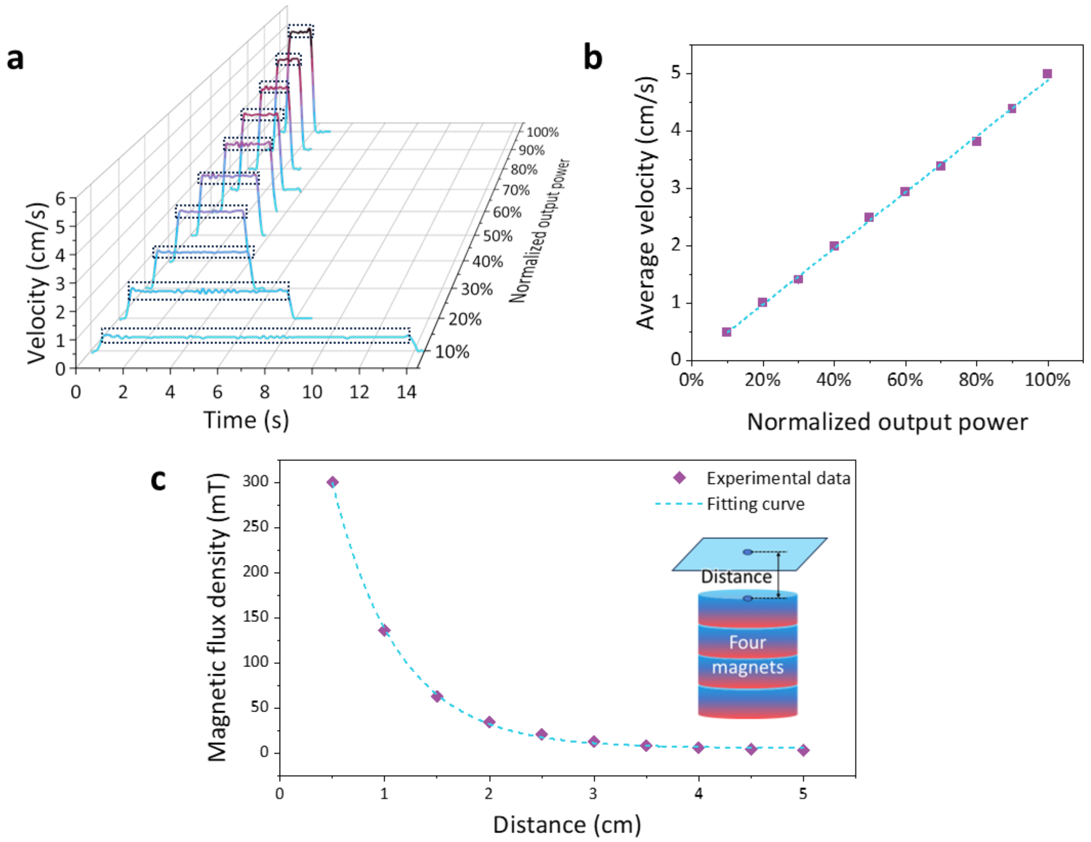

Fig. S29. Measurement for velocity of a robotic arm under different output powers and magnetic flux density under different distances. a, The velocity of the robotic arm under different output powers changes over time. **b**, Output power as a function of the average velocity of robotic arm, where these average velocities are calculated from the data in plateau (black boxes in **a**). **c**, Magnetic flux density versus the distance between a cylindrical permanent magnet (N52, 15 mm in diameter × 20 mm in height) and sample, where the magnet is assembled from four small magnets. The insert depicts the definition for the distance.

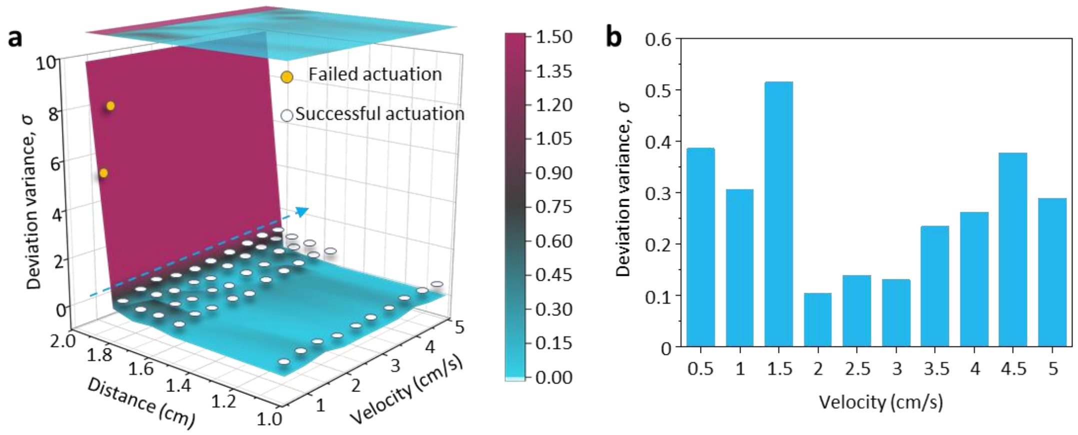

Fig. S30. a, Motion synchronization between HCC and a moving magnet fixed on a robotic arm for actuation, at various actuation distances and under different actuating velocities. **b**, Deviation variances at various actuating velocities when the magnet-MHC distance is maintained to 1.8 cm (blue dotted line in **a**).

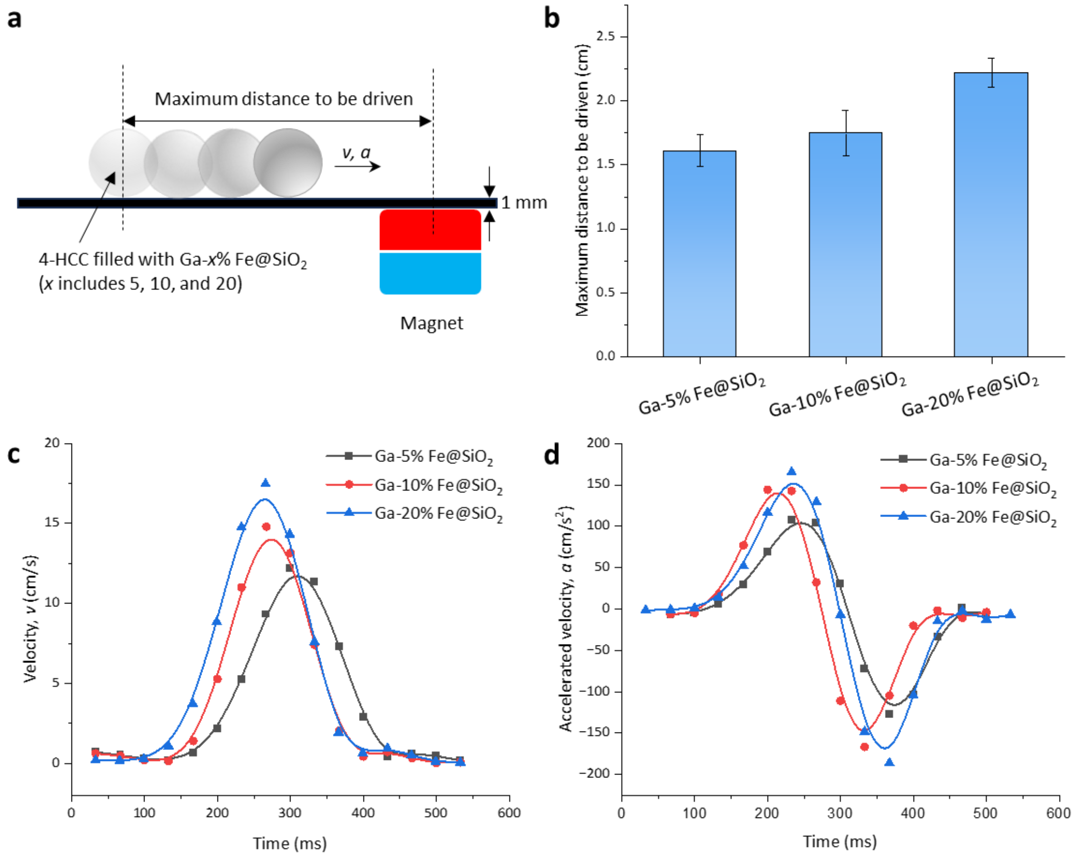

Fig. S31. a, Schematic illustration showing the magnetic field induced actuation process of 4-HCC. **b**, Maximum distance to be driven as function of magnetic particle content in magnetic liquid metals. The variation in velocity *v* (**c**) and accelerated velocity *a* (**d**) over time for Ga-5% Fe@SiO_2, Ga-10% Fe@SiO_2, and Ga-20% Fe@SiO_2 under the same magnetic field.

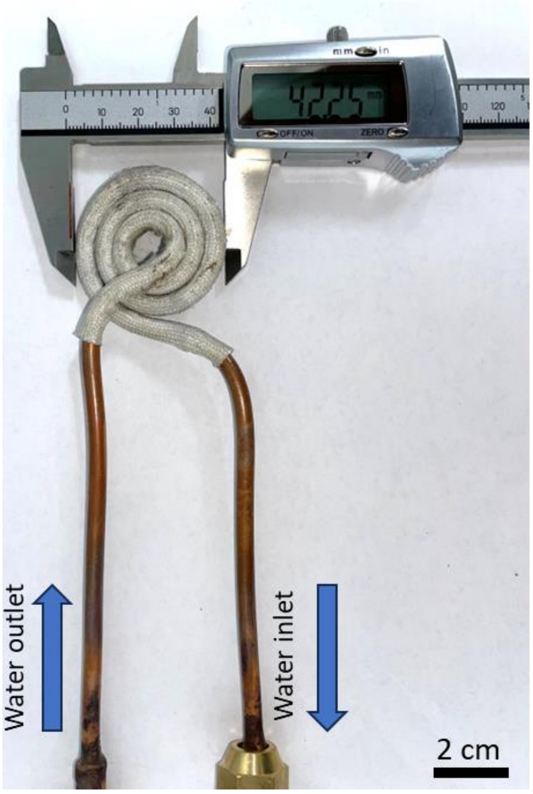

Fig. S32. Optical image of the used electromagnetic coil. A hollow internal structure of the copper electromagnetic coil allows water to flow inside, thus achieving the cooling of the device.

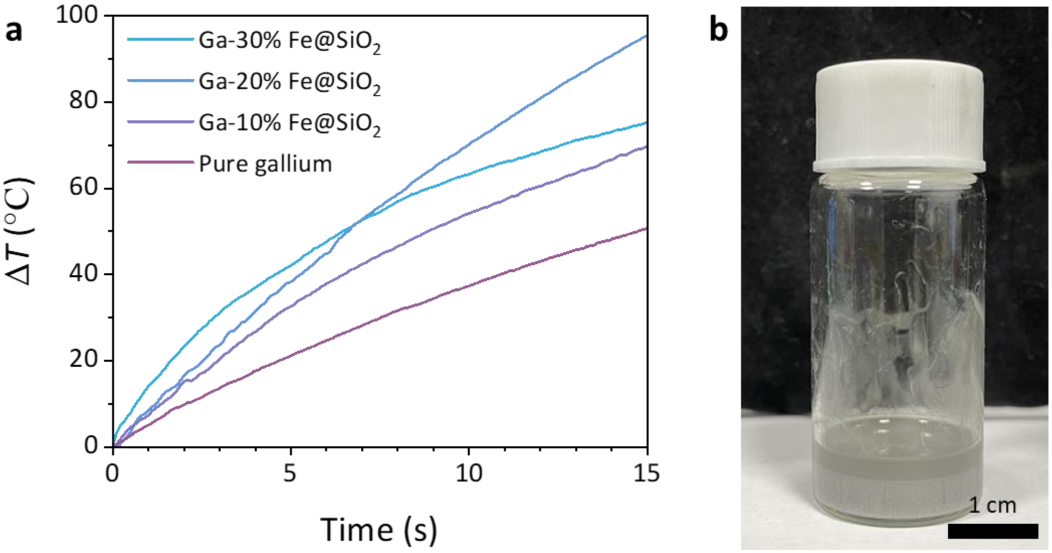

Fig. S33. Wireless induction heating performance for various samples. a, Temperature changes of MLMs with different Fe@SiO_2 mass ratios over time at the same magnet-sample distance (2 cm). **b**, Optical image showing the emulsion of grey Ga-20% Fe@SiO_2, which consists of micro/nano Ga-20% Fe@SiO_2 particles dispersed in an ethyl alcohol solution containing 10wt% PVP.

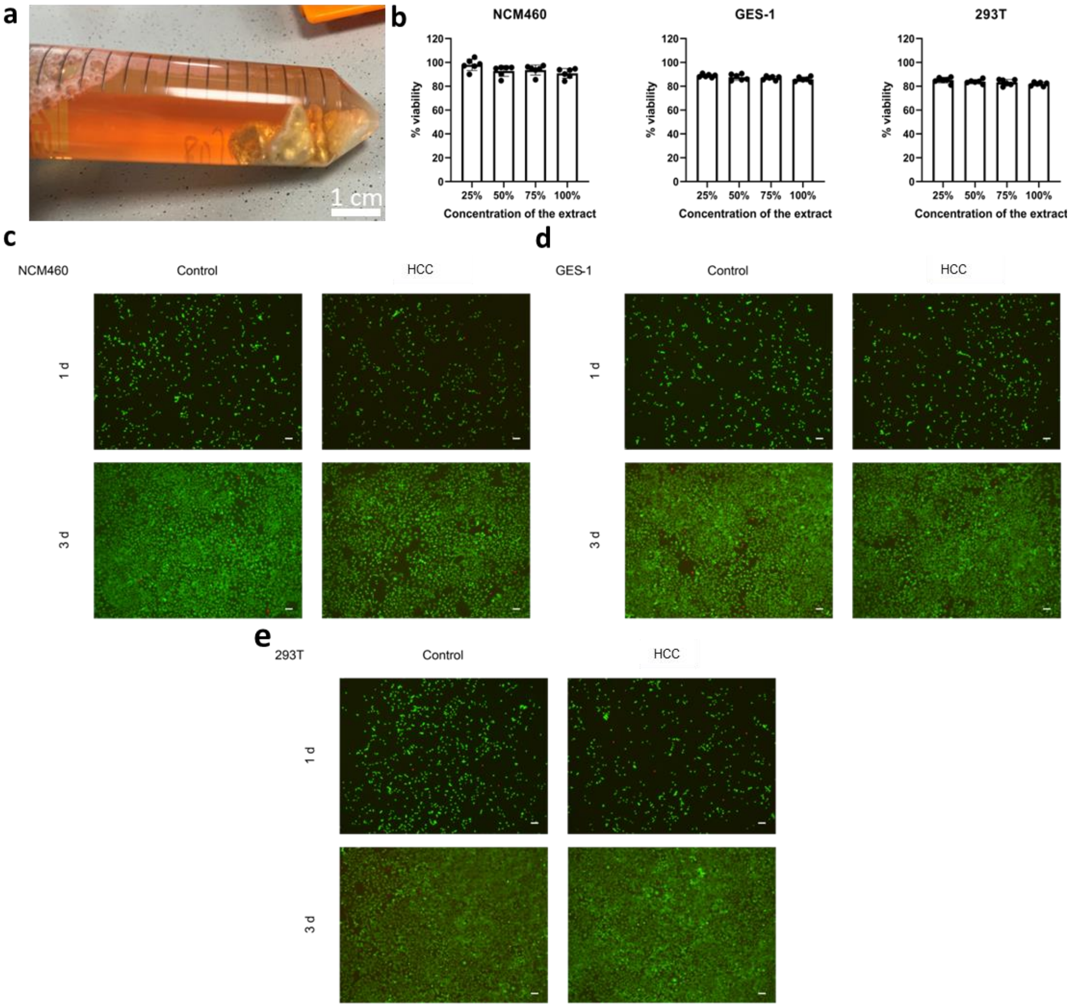

Fig. S34. Viability of various cell lines after 24 h exposure to the leach liquors from HCCs. a, Immersing 4-HCCs in a 37℃ standard medium (Dulbecco's Modified Eagle Medium, DMEM) to obtain leach liquors, where the extraction ratio is 0.2 g/mL. **b**, Cell viability of obtained leach liquors on various normal cells using CCK-8 kit to test. Cell lines are normal human colon mucosal epithelial cells (NCM460), human gastric epithelial cells (GES-1), and human embryonic kidney cells (293T). The leach liquors from HCC further are dissolved in the DMEM to form different concentrations (25%, 50%, 75%, and 100% v/v). Cells are adherently cultured in the mixed DMEM/HCC, and CCK8 kits are used to detect the viability after 24 h. All data are presented as mean ±s.d.; *n* = 6 wells are tested for each treatment per cell line. Viabilities are normalized to untreated cells. **c-e**, Representative confocal microscope images of Live/Dead assay of NCM460 (**c**), GES-1 (**d**) and 293T (**e**) cultured on the control (DMEM) and the leach liquor (100% v/v) from HCC for 24 h and 72 h. Red/green areas represent dead/living cells, respectively. Three independent experiments are conducted with similar results. Scale bar, 100 μm.

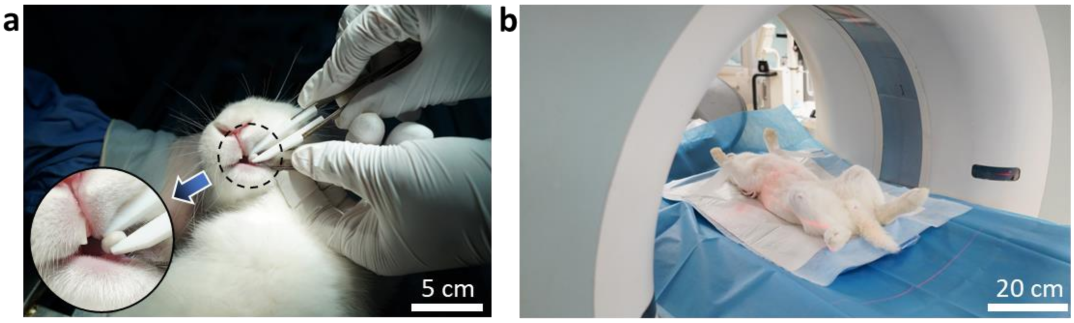

Fig. S35. Oral administration of HCCs for rabbits and medical CT imaging. a, Oral administration for a unanesthetized rabbit using an HCC, where the diameter of capsule is about 6-8 mm to ease swallowing. **b**, Photography showing medical CT scan for an anesthetic rabbit that has swallowed an HCC.

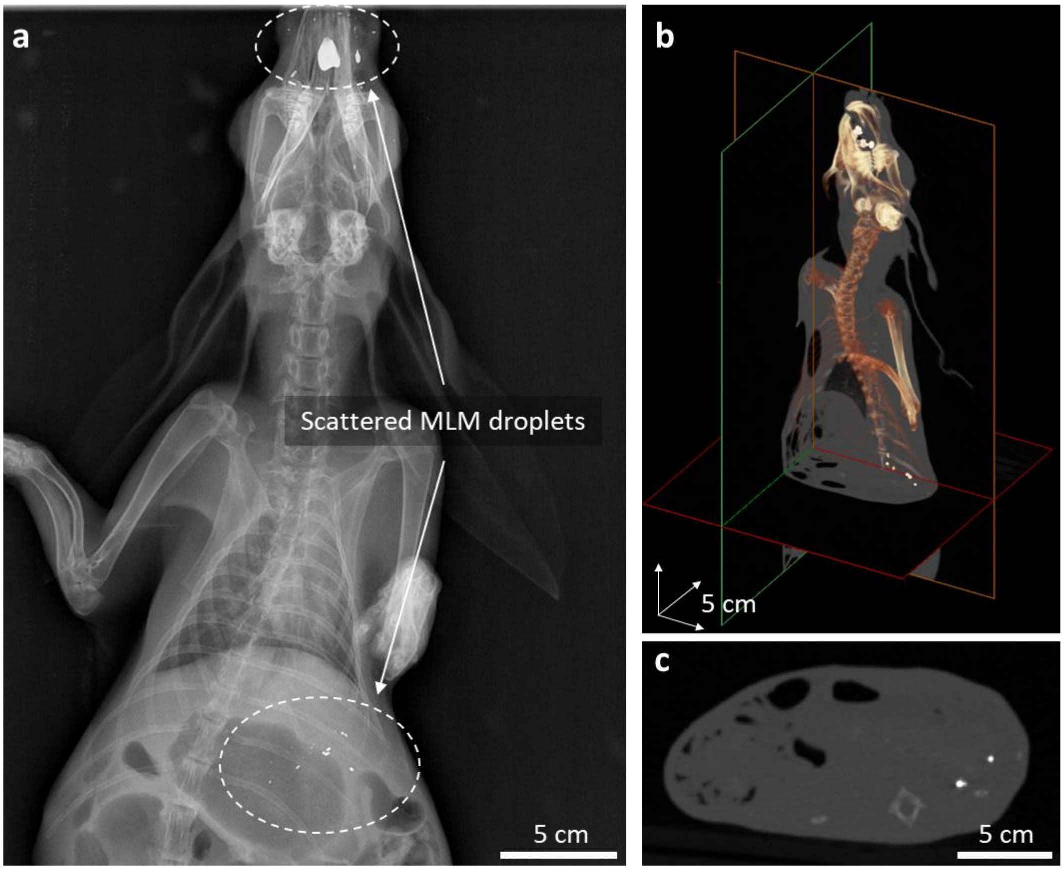

Fig. S36. X-ray detection for control group. a, X-ray image showing a rabbit that swallows bare MLM droplets. **b**, 3D reconstruction image from CT results showing the spatial distribution of scattered MLM droplets in the upper gastrointestinal tract of the control rabbit. **c**, Selected cross-section image from **b** showing the scattered MLM droplets in the rabbit stomach.

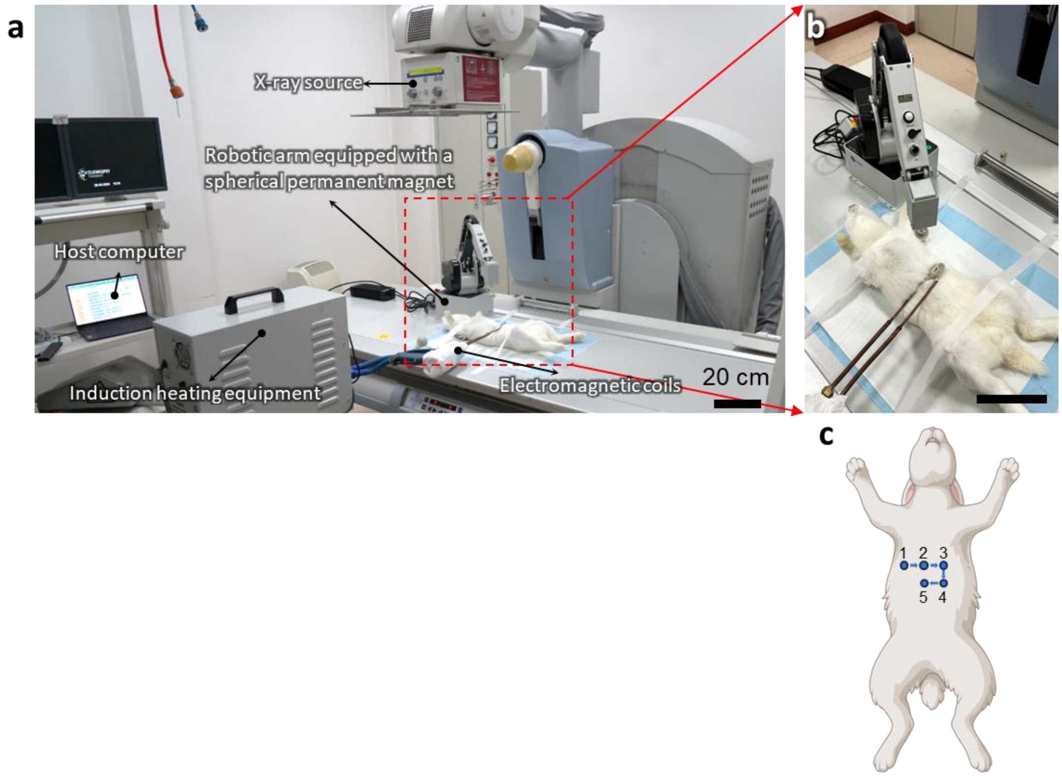

Fig. S37. *In vivo* demonstration for remotely magnetic field-driven manipulation for HCC. a, Overview of equipment for *in vivo* demonstration including the X-ray source for real-time imaging, induction heating equipment, and an electromagnetic coil to generate a high-frequency alternating magnetic field. A robotic arm equipped with a permanent magnet is used to guide the HCC within a rabbit stomach, which is controlled by programming from a host computer. **b**, Zoom-in image of the red box in **a** showing the arrangement for heating equipment and driving component. **c**, Schematic of the pre-programed motion trajectory for the robotic arm to govern the inner HCC.

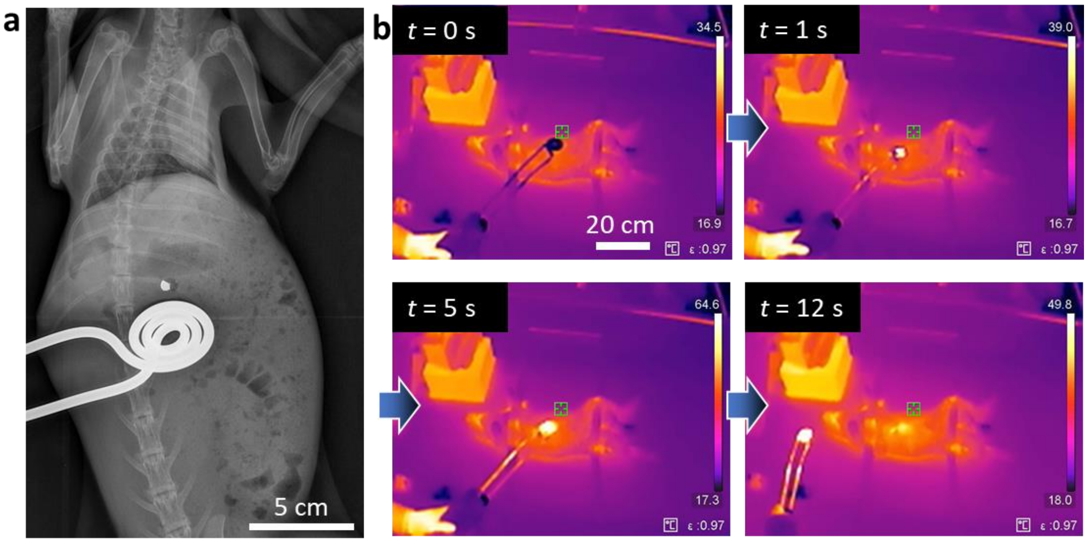

Fig. S38. *In vivo* demonstration for wireless induction heating using HCC. a, X-ray image showing the position of an internal HCC and the external heating devices before induction heating. **b**, The process of *in vivo* targeted thermal ablation. At $t = 0$ s, the electromagnetic coil is placed on a proper position to approach the inner HCC for ease of subsequent effective heating. At $t = 1$ s, the alternating magnetic field is activated to heat the HCC wirelessly. Then, the electromagnetic coil is removed after finishing wireless heating. The above steps are conducted 5 times for repeated heating to ensure the success of thermal ablation operation.

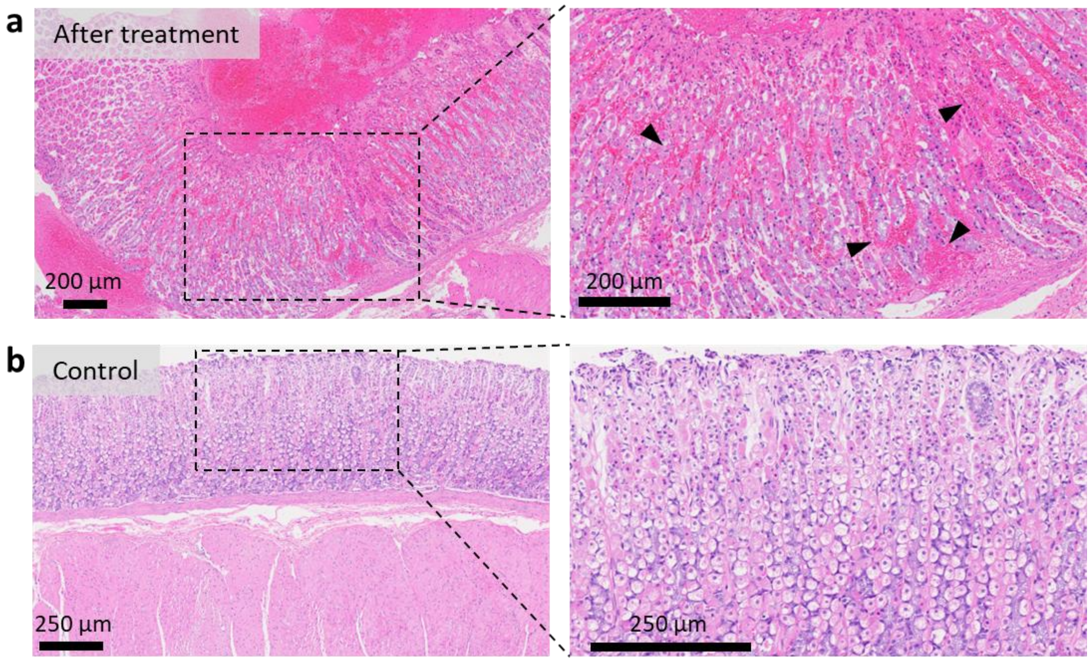

Fig. S39. Representative photomicrographs of H&E in paraffin-embedded scalded gastric tissue from rabbits after thermal ablation operation (**a**) and normal gastric tissue from a control rabbit (**b**). The black triangles indicate the presence of numerous red blood cells in the gastric mucosal layer. In the normal gastric tissue, the gland structure exhibits neither disordered arrangement, nor evident hemorrhagic or erosive lesions.

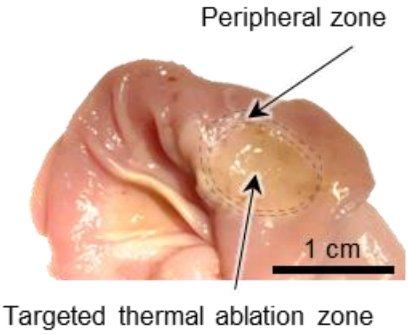

Fig. S40. The positions of the targeted thermal ablation zone and the peripheral zone on scalded gastric tissues from an *in vivo* rabbit model after thermal ablation. During the heating process, peripheral zone is formed by sub-lethal thermal exposure as residual heat diffuses outward. Peripheral zone is often located within a 1 - 2 mm range around the rim of thermal ablation zone.

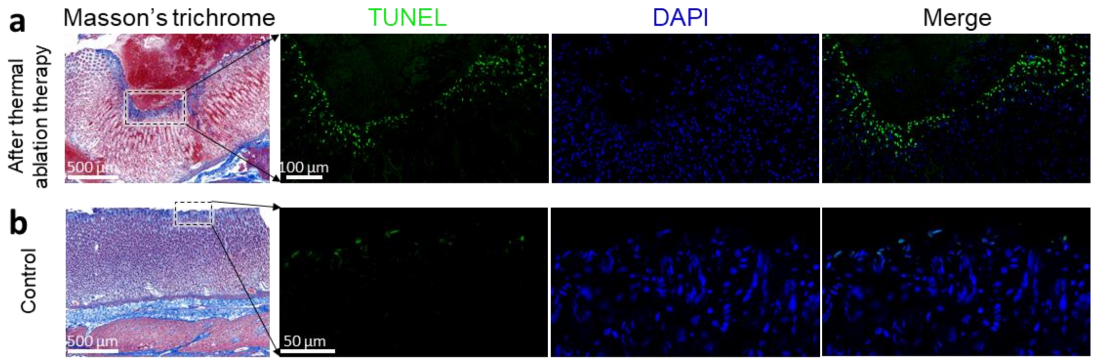

Fig. S41. Representative photomicrographs of Masson's trichrome staining and TUNEL staining from rabbits after thermal ablation operation (**a**) and control groups (**b**). Masson's trichrome staining in **a** outlines the ulcer in the mucosal layer does not penetrate into the submucosa. It means that the damaged gastric mucosa can regenerate after thermal ablation operation, thus avoiding scar fibroplasia. TUNEL staining confirms significant heat-induced apoptosis is afforded by after thermal ablation operation using HCC. Channels are: DAPI nuclear stain (blue) and apoptosis by TUNEL (green).

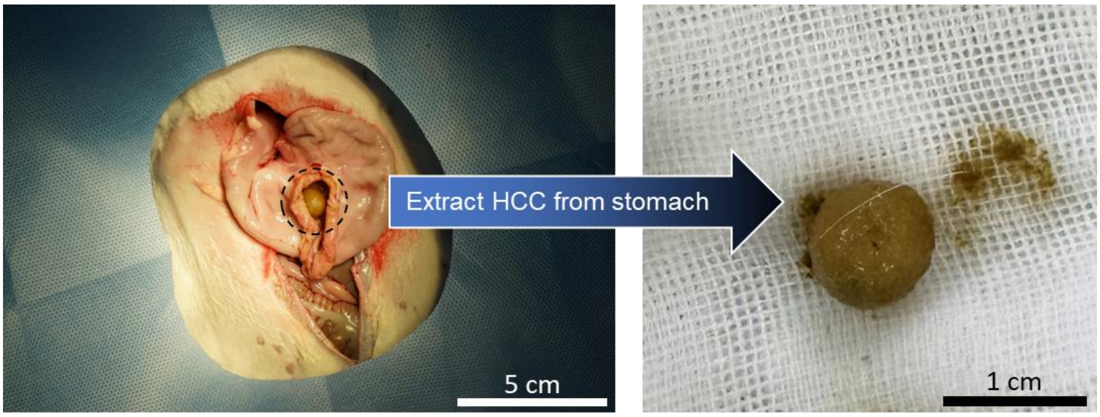

Fig. S42. Optical images showing the structure integrity of the HCC after magnetic manipulations. The color of HCC changes to brown-green due to the mixing of HCC with gastric chyme in rabbit stomach.

References

1. Cao L, Yu D, Xia Z *et al.* Ferromagnetic liquid metal putty‐like material with transformed shape and reconfigurable polarity. *Adv Mater* 2020; **32**: 2000827.

2. Ren L, Sun S, Casillas‐Garcia G *et al.* A liquid‐metal‐based magnetoactive slurry for stimuli‐responsive mechanically adaptive electrodes. *Adv Mater* 2018; **30**: 1802595.

3. Shen Y, Jin D, Li T *et al.* Magnetically responsive gallium-based liquid metal: preparation, property and application. *ACS Nano* 2024; **18**: 20027–54.

4. Osintsev AM, Vasilchenko IL, Rodrigues DB *et al.* Characterization of ferromagnetic composite implants for tumor bed hyperthermia. *IEEE Trans Magn* 2021; **57**: 1–8.

5. Palzer J, Eckstein L, Slabu I *et al.* Iron oxide nanoparticle-based hyperthermia as a treatment option in various gastrointestinal malignancies. *Nanomaterials* 2021; **11**: 3013.

6. Yu Y, Miyako E. Alternating-magnetic-field-mediated wireless manipulations of a liquid metal for therapeutic bioengineering. *Iscience* 2018; **3**: 134–48.

7. Wang H, Chen S, Li H *et al.* A liquid gripper based on phase transitional metallic ferrofluid. *Adv. Funct Mater* 2021; **31**: 2100274.

8. Gao J-Y, Zhang X-D, Fu J-H *et al.* Numerical investigation on integrated thermal management via liquid convection and phase change in packed bed of spherical low melting point metal macrocapsules. *Int J Heat Mass Tran* 2020; **150**: 119366.

9. Yu D-H, He Z-Z. Shape-remodeled macrocapsule of phase change materials for thermal energy storage and thermal management. *Appl Energ* 2019; **247**: 503–16.

10. Yao X, Liu J, Yang C *et al.* Hydrogel paint. *Adv Mater* 2019; **31**: 1903062.

11. Computational wrapping: A universal method to wrap 3D-curved surfaces with nonstretchable materials for conformal devices. *Sci Adv* 2020; 6, eaax6212.

12. Chen R, Xiong Q, Song R *et al.* Magnetically controllable liquid metal marbles. *Adv Mater Interfaces* 2019; **6**: 1901057.

13. Sun J-Y, Zhao X, Illeperuma WRK *et al.* Highly stretchable and tough hydrogels. *Nature* 2012; **489**: 133–6.

14. Bai R, Chen B, Yang J *et al.* Tearing a hydrogel of complex rheology. *J Mech Phys Solids* 2019; **125**: 749–61.
